# Suppression of Somatic Expansion Delays the Onset of Pathophysiology in a Mouse Model of Huntington’s Disease

**DOI:** 10.1371/journal.pgen.1005267

**Published:** 2015-08-06

**Authors:** Helen Budworth, Faye R. Harris, Paul Williams, Do Yup Lee, Amy Holt, Jens Pahnke, Bartosz Szczesny, Karina Acevedo-Torres, Sylvette Ayala-Peña, Cynthia T. McMurray

**Affiliations:** 1 Life Sciences Division, Lawrence Berkeley National Laboratory, Berkeley, California, United States of America; 2 Department of Molecular Pharmacology and Experimental Therapeutics, Mayo Clinic, Rochester, Minnesota, United States of America; 3 Department of Bio and Fermentation Convergence Technology, Kookmin University, Seoul, Korea; 4 Department of Neuropathology, University of Oslo, Oslo, Norway; 5 LIED, University of Lübeck, Lübeck, Germany; 6 Department of Anesthesiology, The University of Texas Medical Branch, Galveston, Texas, United States of America; 7 Puerto Rico Center for Inherited Diseases, University of Puerto Rico, San Juan, Puerto Rico; 8 Department of Pharmacology and Toxicology, University of Puerto Rico, San Juan, Puerto Rico; St Jude Children's Research Hospital, UNITED STATES

## Abstract

Huntington’s Disease (HD) is caused by inheritance of a single disease-length allele harboring an expanded CAG repeat, which continues to expand in somatic tissues with age. The inherited disease allele expresses a toxic protein, and whether further somatic expansion adds to toxicity is unknown. We have created an HD mouse model that resolves the effects of the inherited and somatic expansions. We show here that suppressing somatic expansion substantially delays the onset of disease in littermates that inherit the same disease-length allele. Furthermore, a pharmacological inhibitor, XJB-5-131, inhibits the lengthening of the repeat tracks, and correlates with rescue of motor decline in these animals. The results provide evidence that pharmacological approaches to offset disease progression are possible.

## Author Summary

Huntington’s Disease (HD) is caused by inheritance of a single disease-length allele harboring an expanded CAG repeat, which continues to expand in somatic tissues with age. There is no correction for the inherited mutation, but if somatic expansion contributes to disease, then a therapeutic approach is possible. The inherited disease allele expresses a toxic protein, and whether further somatic expansion adds to toxicity is unknown. Here we describe a mouse model of Huntington’s disease that allows us to separate out the effects of the inherited gene from the expansion that occurs during life. We find that blocking the continued expansion of the gene causes a delay in onset of symptoms. This result opens the doors to future therapeutics designed to shorten the repeat.

## Introduction

HD is an autosomal dominant neurodegenerative disorder in which the underlying mutation is a CAG expansion within exon 1 of the mutant allele [1–3]. Inheriting the expanded HD allele is sufficient to develop disease. However, somatic expansion is prominent in HD patients and it has been speculated, but remains controversial, as to whether the somatic expansion contributes significantly to the pathophysiology. Although the length of the CAG expansion correlates with toxicity, there is as yet no direct evidence that suppressing further somatic expansion will be beneficial, since the toxic protein from the inherited allele is also expressed [4–10]. There is intense interest in determining whether blocking somatic expansion is a viable therapeutic option [1–3, 11–13], yet testing the hypothesis in humans has been exceptionally difficult for at least three reasons.

First, human brain tissue is available only postmortem. Thus, it has not been possible to link somatic expansions with HD progression. Analysis of postmortem brain from a cohort of HD patients infers a relationship between length and phenotype [11–13]. However, because somatic expansion changes with age, the lengths of the repeat tracts after death are not the same as those that are present at onset, which occurs decades earlier. Second, the relationship between the inherited repeat length and disease onset in HD is highly variable (S1A Fig)[14]. Indeed, an inherited repeat length among HD patients can predict the average age of onset, but two individual patients with the same inherited tract length can vary as much as 4-fold in the age of onset (between 18 and 80 years) (S1A Fig)[14]. Somatic CAG instability generates a wide distribution of repeat tracts in every patient, making it difficult to link pathophysiology to particular expansion size [4, 5, 7, 9, 10]. Third, and perhaps most important, the inherited repeat tract has its own toxic effects, and whether further somatic expansion adds to toxicity is difficult to determine, even if somatic expansion is prominent. Collectively, the idea that somatic expansion promotes disease is an attractive one, but the inability to resolve the effects of the inherited and somatic repeats renders the relationship a speculation.

These difficulties underscore the value of the mouse models. Age-dependent somatic expansion is well documented in tissues of aging mice expressing the mutant huntingtin protein (mHTT) [15–18], and can be quantified during life (S1B Fig). Nevertheless, animal models suffer from the same difficulties, as do their human counterparts. Specifically, somatic expansion occurs as disease progresses, but the effects of the inherited and somatic expansion are not separable.

**Fig 1:**
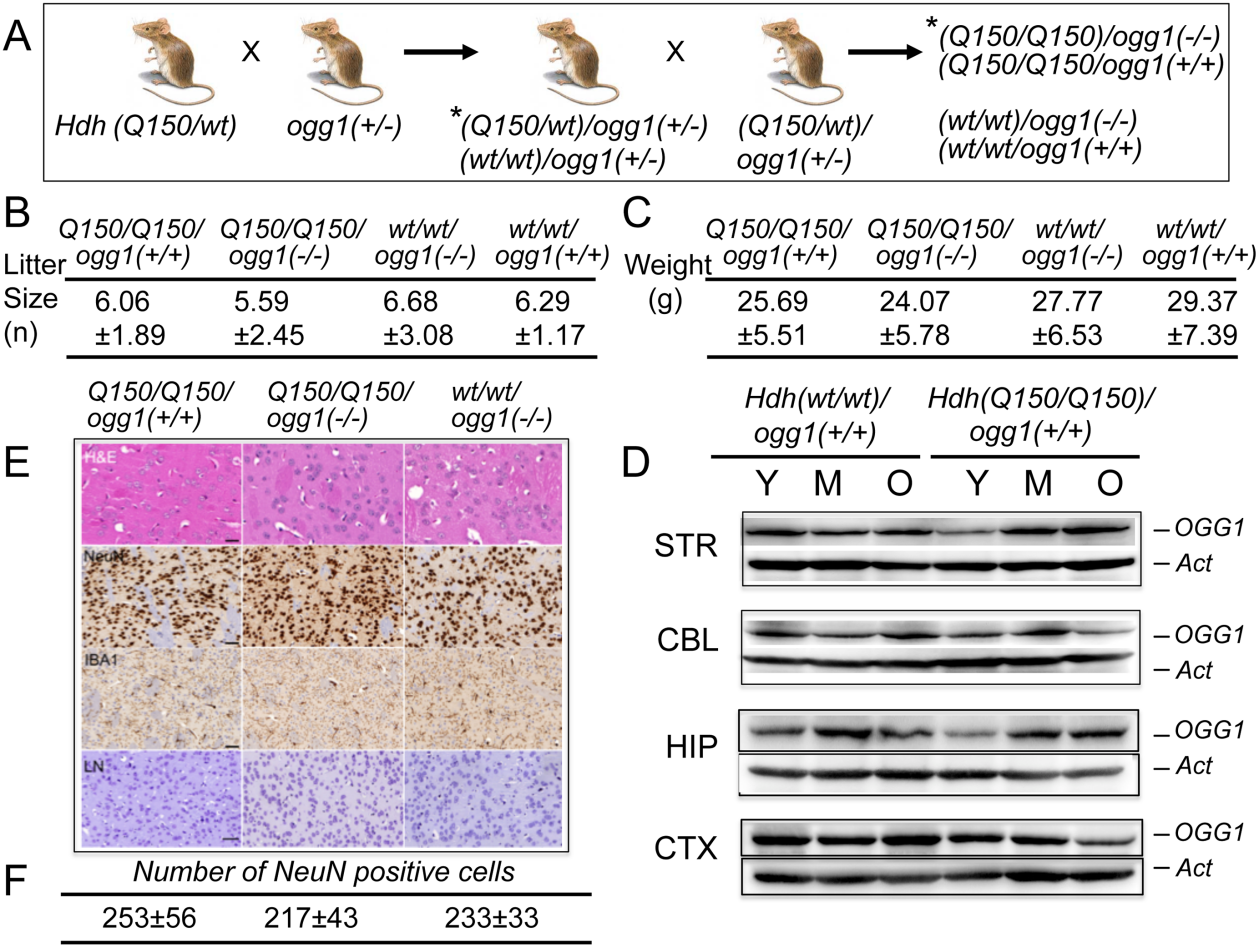
*Hdh(Q150/Q150)/ogg1(+/+)* and *Hdh(Q150/Q150)/ogg1(-/-)* animals are similar by physiological criteria. (A) Schematic of crosses between *Hdh(Q150/wt)* and *ogg1(+/-)*; (*) only a subset of the resulting genotypes from the breeding step are shown. The OGG1 (B) The average litter size for *Hdh(Q150/Q150)ogg1(+/+)*, *Hdh(Q150/Q150)ogg1(-/-)*, *Hdh(wt/wt)/ogg1(-/-)* and *Hdh(wt/wt)/ogg1(+/+) genotypes*. (C) The average weight (grams) for *Hdh(Q150/Q150)ogg1(+/+)*, *Hdh(Q150/Q150)ogg1(-/-)*, *Hdh(wt/wt)/ogg1(-/-)* and *Hdh(wt/wt)/ogg1(+/+)* animals at 25 weeks. A full table of weights and litter sizes for all nine genotypes are presented in S1 Table (in S2B Fig). (D) OGG1 resolved on an SDS-PAGE gel migrates as a 41 kDa (368 aa) protein. The age-dependence of OGG1 protein expression relative to actin controls: Y is 7–10 weeks, M is 12–16 weeks; O is greater than 30 weeks. The brain regions are; STR is striatum, CBL is cerebellum, HIP is hippocampus, CTX is cortex, as indicated. (E) Histological analysis of brain slices (caudate-putamen) from *Hdh(Q150/Q150)ogg1(+/+)*, *Hdh(Q150/Q150)ogg1(-/-)*, and *Hdh(wt/wt)/ogg1(-/-)* at 7–16 weeks. H&E is Hematoxylin and Eosin, which visualize protein and nucleic acid-rich regions, NeuN detects neurons, IBA1 is ionized calcium binding adaptor molecule 1, which detects microgliosis, and LN (Luxol-Nissl) stain detects overall cellular pattern and morphology. Scale bar is 50μm. Quantification of neurons by NeuN staining comprised 3 animals, 5–10 tissues slices and 10 random fields on each slice. (F) Quantification of the number of NeuN positive cells from digital images of brain slices of each of the three indicated genotypes. Shown are the histological analysis for only three genotypes that are most likely to exhibit pathophysiology. None of the genotypes displayed differences relative to controls.

We have created a novel mouse model in which the effects of the inherited and somatic expansion are resolved in the same genetic background. We previously reported that the 7,8-dihydro-8-oxo-guanine (8-oxo-G) glycosylase (OGG1) is not essential for life, but its role in base excision repair of oxidative DNA damage causes genetic instability at CAG repeats in *R6/1* mice harboring a toxic truncated mHTT fragment [19] (S1C Fig, **A Toxic Oxidation Cycle**). We created a more physiological model by crossing *Hdh(Q150/wt)* heterozygous “knock-in” mice [20], harboring disease-length CAG repeats knocked into the mouse Huntingtin locus, with *ogg1(+/-)* [21] heterozygous knockout mice. The *Hdh(Q150)* mouse line was chosen because it is a late onset model with a wide window to observe the earliest expansions and their relationship to the onset of early phenotypes. The cross produced nine genotypes that expressed all combinations of wt and the expanded full-length mutant HD allele with a normal, a reduced gene complement or entirely lacking OGG1 (Fig 1A and S2A Fig). We report here that loss of somatic expansion in the *Hdh(Q150/Q150)/ogg1(-/-)* crosses delays the onset of disease by around 7–10 months relative to their *Hdh(Q150/Q150)/ogg1(+/+)* littermates, although they both inherit a similar disease-length HD allele. We further demonstrate that a pharmacological agent, which reduces the DNA substrates for OGG1, also reduces instability. Thus, blocking somatic expansion is beneficial, providing a therapeutic avenue for treating these deadly diseases.

## Results

### Loss of OGG1 does not alter measured physiological properties of the *Hdh* mice

Loss of OGG1 in *Hdh(Q150/wt)/ogg1(-/-)* or *Hdh(Q150/Q150)/ogg1(-/-)* mice had no effect on the survival or fertility of these animals relative to the controls, and the average weight of animals from all genotypes did not significantly differ (Fig 1 and S2B Fig). Litter sizes were consistent, with roughly equal numbers of male and female progeny independent of age (P = 0.31), OGG1 genotype (P = 0.26) or HD genotype (P = 0.40) (Fig 1 and S2B Fig). All nine genotypes were well groomed and indistinguishable by visual appearance and weight (Fig 1C, shown are only four genotypes)(S2B Fig). We were also unable to detect morphological abnormalities (H&E, Nissl) or microgliosis (IBA1) in the caudate-putamen of *Hdh(Q150/Q150)/ogg1(+/+)* and *Hdh(Q150/Q150)/ogg1(-/-)* animals or controls within the first 8 months of life (Fig 1E). There were no inclusions in any genotype detected by ubiquitin staining within the first 40 weeks of life. Similarly, there was no loss of neurons during that period, as detected by NeuN staining patterns shown for *Hdh(wt/wt)*, *Hdh(Q150/Q150)/ogg1(-/-)* and *Hdh(Q150/Q150)/ogg1(+/+)* littermates (Fig 1E and 1F).

**Fig 2:**
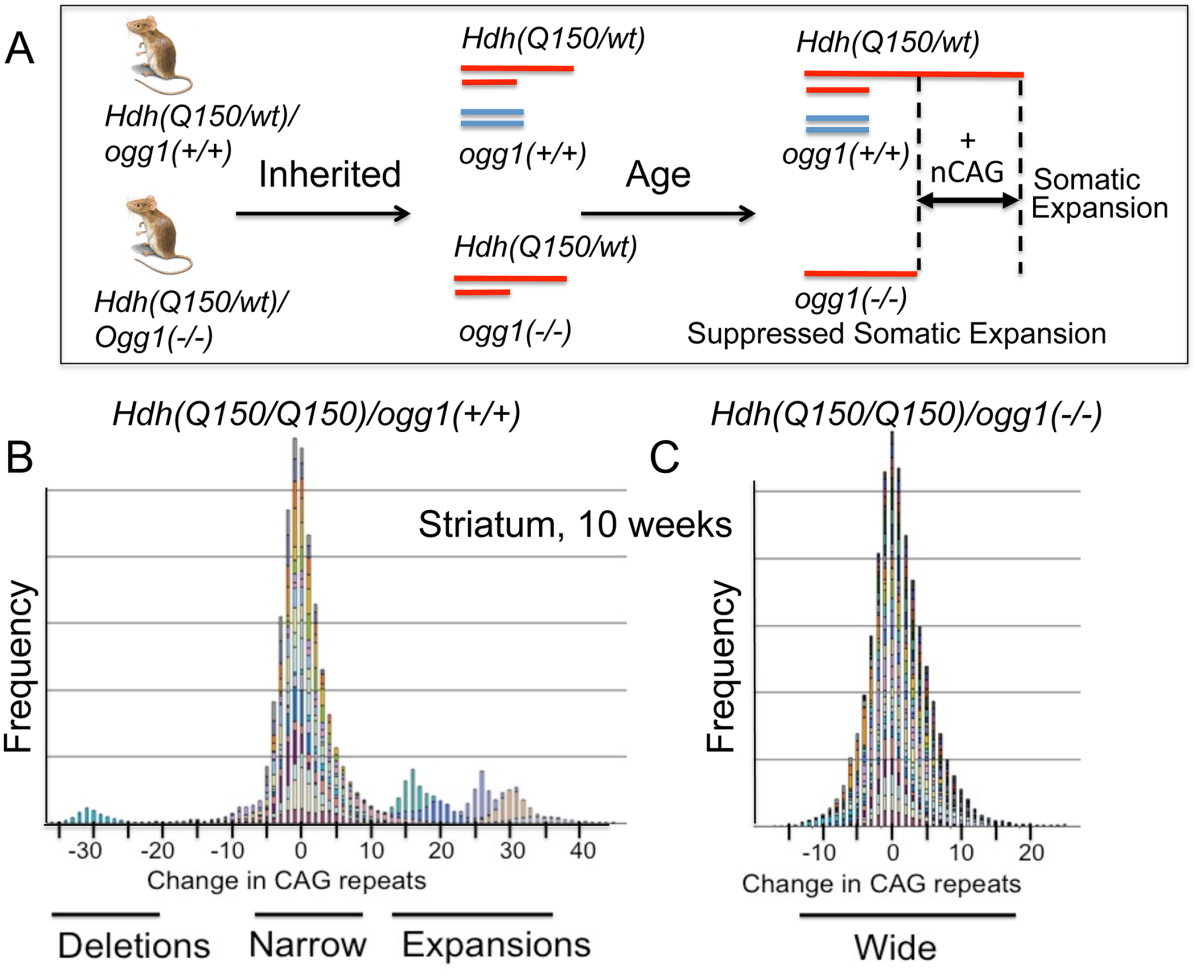
Loss of OGG1 suppresses the average repeat length in *Hdh(Q150/Q150)/ogg1(-/-)* animals. (A) Schematic representation of the experiment, as described in text. The red lines depict the *Hdh* alleles in a heterozygous *Hdh* animals that do *Hdh(Q150/Q150)/ogg1(+/+)* or do not *(Q150/wt)/ogg1(-/-)* express OGG1. The blue lines depict the *ogg1* alleles and no blue lines indicate their absence in *Hdh(Q150/wt)/ogg1(-/-)* mice. The absence of OGG1 in the *Hdh(Q150/wt)/ogg1(-/-)* suppresses age-dependent somatic expansion (+CAG) that is observed in the *Hdh(Q150)* allele of *Hdh(Q150/wt)/ogg1(+/+)* animals. The increased length of the red line represents somatic expansion the long, disease-length allele. (B) The stacked bar graph is a frequency plot for pooled normalized repeat tracts from a representative set of animals for illustration purposes (n = 6) (B) *Hdh(Q150/Q150)/ogg1(+/+)* and (C) *Hdh(Q150/Q150)/ogg1(-/-)* animals in the striatum at 10 weeks to demonstrate the asymmetry of the distributions, as indicated. Colors represent individual mice. CAG repeats at HD locus were amplified and analyzed as described previously [19]. Data were analyzed using GeneMapper software v4.

We did not observe global age-dependent differences in expression of OGG1 or mHTT in the brains of *Hdh(wt/wt)* or *Hdh(Q150/Q150)* animals between 7 and 60 weeks (Fig 1D and S3 Fig). Thus, these animals expressed a relatively constant ratio of HTT/mHTT and OGG1 throughout their life. The properties of the *ogg1(-/-)* mouse have been investigated for more than a decade [21]. OGG1 acts on DNA as a repair enzyme that preferentially removes oxidized bases such as 8-oxoGuanine (8-oxo-G) [1, 19]. Mice lacking OGG1 have no obvious phenotype for most of their life [21–23]. Each mouse line was isogenic, and consequently free from additional genetic modifiers that confound the analysis of humans [24–29]. By all criteria, we observed no physiological differences between *Hdh(Q150/Q150)/ogg1(-/-)* mice and their *Hdh(Q150/Q150)/ogg1(+/+)* littermates at early ages, other than differences in their somatic CAG tracts. Therefore, comparing the onset of toxicity between littermates of these two genotypes provided a suitable system for resolving the impacts of the inherited and somatic expansion in a physiologically relevant model (Fig 2A).

### The size of the inherited alleles did not influence the size of the somatic expansion

We tested whether somatic expansion in the brain contributed to the onset of toxicity in a group of roughly 1200 animals. Each genotype was divided into 7 age groups (roughly 16–24 animals per group), which were separated by 5 or 10-week intervals at early ages (5-10wks, 11-20wks, 21-30wks, 31-40wks), and 20-week or 40-week intervals at later ages (61-80wks and 81-120wks). Six genotypes were the focus of the analysis; *Hdh(Q150/wt)/ogg1(+/+)*, *Hdh(Q150/Q150)/ogg1(+/+)*, *Hdh(Q150/wt)/ogg1(-/-)*, *Hdh(Q150/Q150)/ogg1(-/-)*, *Hdh(wt/wt)/ogg1(-/-)*, and *Hdh(wt/wt)/ogg1(+/+)* (wild-type). Animals were tested for motor function (a 5-day testing period) at a specified age, and immediately sacrificed for DNA analysis of their CAG tract length after testing. This protocol eliminated any learning bias due to repeated testing of the same animals over the 100-week period. Moreover, each age group comprised independent animals with an equal number of males and females to create random populations for testing. The premise was to increase power of analysis, and to reflect the general properties of aging animals rather than a specific group of animals with age.

The size distribution of CAG repeats was established using Genescan [30], a rapid PCR-based method, which provides an immediate indication of whether expansion has occurred and the most prevalent sizes. The size at birth was on average around 117 repeats and formed a single narrow Gaussian distribution, whose midpoint was taken as the inherited repeat size. The distribution of inherited repeats was variable among animals (a standard deviation ±12 repeats). The bulk of the inherited alleles were within 24 repeats of each other, but a maximum of 48 repeats separated the smallest and largest inherited alleles (± 2SD). The number of inherited repeats in these animals, however, had no influence on somatic tract length in the hippocampus (HIP) (P = 0.75), cortex (CTX) (P = 0.26), and cerebellum (CBL) (P = 0.59), and when averaged over all four brain regions (P = 0.95). Overall, there was little selective advantage for the inherited allele to expand among *Hdh(Q150/wt)* and *Hdh(Q150/Q150)* genotypes in any of the brain regions tested, and the changes in their age-dependent somatic tracts could be directly compared.

### Absence of OGG1 suppresses somatic expansion

To compare the age-dependent somatic changes among age groups and genotypes, we normalized the changes in repeat tract length by subtracting the CAG tracts measured at birth from the CAG tracts measured at the age of interest. The distributions were expressed as the change in repeat length and summed from all animals within an age group to create a single global distribution that characterized the population. Using these global distributions, somatic expansions in the striatum, hippocampus, cerebellum, cortex, or all regions combined, were a measure of the overall age-dependent changes in repeat length in each genotype. The focus was primarily on the age-dependent changes that occurred between birth and 40 weeks, since pathophysiology in the *Hdh(Q150/Q150)* line develops within that time frame [20].

Instability occurred with age in the disease-length allele in all genotypes in all four regions of the brain (S4 Fig). When measured at the corresponding age for onset of motor symptoms, the changes were small, and the length distributions were heterogeneous (Fig 2B, a representative illustration). For example, at 10 weeks (Fig 2B and 2C), the mean number of somatic CAG repeat changes in the striatum (STR) were 4.89±1.41 and 2.04±1.13 in *Hdh(Q150/Q150)/ogg1(+/+)* and *Hdh(Q150/Q150)/ogg1(-/-)* animals, respectively, but the number of extreme changes in the expansions fell within +2σ and +3σ from the mean (S5A Fig).

**Fig 3:**
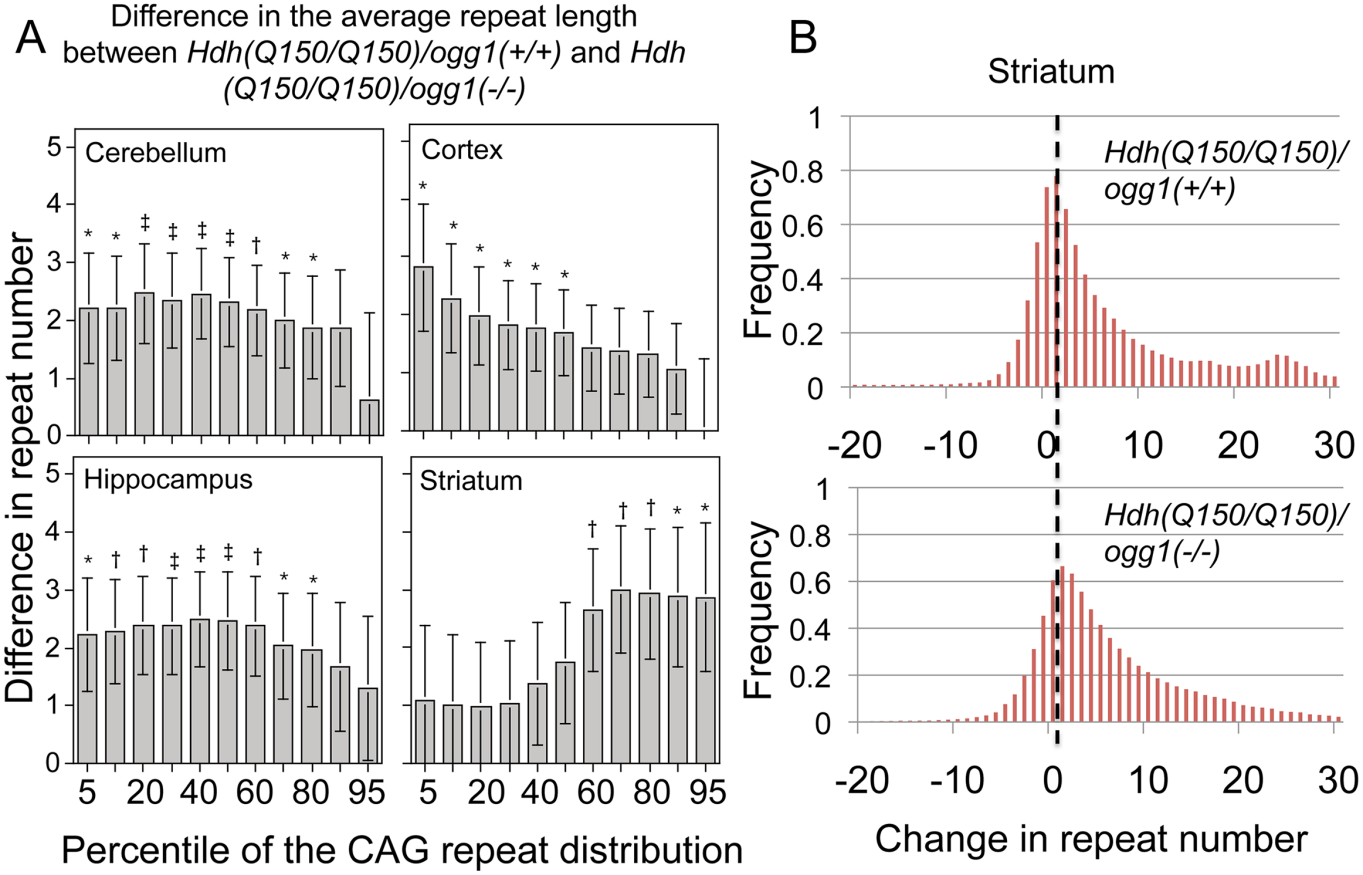
The distribution of CAG tract suppression by OGG1 is region-specific and affects tract sizes across the entire asymmetric distribution. (A) Analyzed were the pooled distributions from the entire set of somatic changes in each of the *Hdh(Q150/Q150)/ogg1(+/+)* and *Hdh(Q150/Q150)/ogg1(-/-)* animals between birth and 40 weeks *(N = 160–200 per genotype)*. Each cell is the difference in size between the *Hdh(Q150/Q150)/ogg1(+/+)* and *Hdh(Q150/Q150)/ogg1(-/-)* genotypes in the segmented quantiles, from 1 to 99. The differences along the asymmetric distributions indicate the sizes of the somatic expansions that were suppressed by OGG1 in each of the indicated brain regions. The changes in CAG tract length were not statistically different in animals that harbored one or two *Hdh(Q150)* alleles and so they were pooled in each distribution to increase the power of the analyses. Bracketed lines represent 1 SE. Significance levels coded: * P≤0.05; † P≤0.01; ‡ P≤0.005. (B) Examples of pooled distributions in striatum over 40 weeks, illustrating the altered CAG repeat tract lengths.

Despite the inherent variability, the effects of the loss of OGG1 were evident. Loss of OGG1 in most regions of the brain resulted in a modest, but significant reduction in repeat tract length in *Hdh(Q150/Q150)/ogg1(-/-)* and *Hdh(Q150/wt)/ogg1(-/-)* relative to *Hdh(Q150/Q150)/ogg1(+/+)* and *Hdh(Q150/wt)/ogg1(+/+)* animals in the hippocampus (2.20±0.87, P = 0.01), cerebellum (2.10±0.81, P = 0.01), striatum (1.93±1.02, P = 0.06), and cortex (1.55±0.77, P = 0.05), or when all brain regions were pooled (1.82±0.75, P = 02). Due to the asymmetric and wide distribution of the repeat tract changes (S1B Fig), the differences in averages were small. However, a quantile-based statistical approach across the entire distribution provided better insight into the size of the repeat tracts that were suppressed by loss of OGG1. The global distribution of repeat lengths were divided into 1^st^, 5^th^, 10^th^, 20^th^, 30^th^, 40^th^, 50^th^, 60^th^, 70^th^, 80^th^, 90^th^, 95^th^, and 99^th^ percentiles (referred to as cells) (Fig 3A), and we subtracted the mean difference in each cell between *Hdh(Q150/Q150)/ogg1(-/-)* and *Hdh(Q150/Q150)/ogg1(+/+)* genotypes to determine the size of the tracts that were changed (Fig 3A). The analysis was based on the premise that differences between the distributions of the two genotypes were the somatic expansions that were suppressed by loss of OGG1 (Fig 2A). To maximize statistical power, analyses were performed for *Hdh(Q150/Q150)* and *Hdh(Q150/wt)* combined and adjusted for HD zygocity. This was allowed because there was no significant interaction between the effects of HD zygocity and OGG1 knockout status on the number of somatic repeats (P≥0.19).

For the cortex of *Hdh(Q150/Q150)* animals, 90% of the somatic changes occurred in the lower 30^th^ percentile of the distribution (Fig 3A), i.e., loss of OGG1 altered the smallest tract sizes in that region of the brain (Fig 3A). In the hippocampus and cerebellum, the altered tracts were longer with 80% of the expansions spread over short and intermediate lengths (Fig 3A). In contrast to the other brain regions, the most affected somatic lengths in the striatum occurred in the upper 60^th^ percentile of the distribution (Fig 3A). Collectively, the results indicated that *Hdh(Q150/Q150)/ogg1(+/+)* and *Hdh(Q150/Q150)/ogg1(-/-)* animals inherited a similar allele length. However, the age-dependent somatic expansions were larger in *Hdh(Q150/Q150)/ogg1(+/+)* animals (Fig 2B) and were evident in the differences in the integrated distributions (Fig 3A and 3B).

To determine the time window in which the somatic expansions were most significant, we compared the average length of the somatic repeat tracts in each age group when all brain regions were combined (Table 1). The analysis was based on the premise that the difference between the expansions in *Hdh(Q150/Q150)/ogg1(+/+)* and *Hdh(Q150/Q150)/ogg1(-/-)* animals reflected the age where the effects of the somatic and the inherited repeat would be best resolved. When all brain regions were pooled, the mean difference in tract length was elevated 5-fold in *Hdh(Q150/Q150)/ogg1(+/+)* animals relative to *Hdh(Q150/Q150)/ogg1(-/-)*, independently of whether the *Hdh(Q150/Q150)* alleles were measured alone or together with *Hdh(Q150/wt)* animals (Table 1). The average difference between *Hdh(Q150/Q150)/ogg1(+/+)* relative to the *Hdh(Q150/Q150)/ogg1(-/-)* littermates was greatest between 5–20 weeks (Table 1). That is, loss of OGG1 led to an average 5-fold reduction in somatic expansion between 5–10 weeks (5.10±2.53 versus 1.35±0.18 somatic repeats) and about 2-fold at 20 weeks (5.35±1.80 vs. 3.05±0.32 repeats). Few differences in the average change were observed between 30 (3.89±0.28 vs. 3.68±0.65 repeats) and 40 weeks (4.95±0.97 vs. 4.27±0.41 repeats) (Table 1), although there were changes along the length distribution at any age. Collectively, quantifying the size and dynamics of somatic expansion revealed that both *Hdh(Q150/Q150)/ogg1(+/+)* and *Hdh(Q150/Q150)/ogg1(-/-)* littermates inherited the same disease-length allele, but somatic expansion was suppressed in the latter. The somatic expansion was best resolved from the inherited repeats during the first 7 months of life (below 40 weeks).

**Table 1:** The average number of somatic CAG repeat changes in *Hdh(Q150/Q150) and Hdh(Q150/wt)* animals by age and OGG1 knockout status.

Combined *Hdh(Q150/Q150)* and *Hdh(Q150/wt)* alleles^a^		
Weeks	*ogg1(+/+)* ^c^	*ogg1(-/-)* ^d^
10	5.10±2.53	1.34±0.18
20	5.35±1.80	3.05±0.32
30	3.89±0.28	3.68±0.65
40	4.95±0.97	4.27±0.41
Unadjusted means ± standard deviation (SE)		
*Hdh(Q150/Q150)* alone^b^		
Weeks	*ogg1(+/+)* ^e^	*ogg1(-/-)* ^f^
10	4.68±2.79	1.25±0.26
20	5.48±2.09	3.37±0.57
30	3.17±0.44	2.59±1.52
40	4.47±1.70	5.19±0.42
Unadjusted means ± standard deviation (SE)		

^a.^ Unadjusted means ± SE of somatic repeats as a function of age in *Hdh(Q150/Q150)/ogg1(+/+)* and *Hdh(Q150/wt)/ogg1(+/+)* genotypes combined or *Hdh(Q150/Q150)/ogg1(-/-)* and *Hdh(Q150/wt)/ogg1(-/-)* genotypes combined.

^b.^ Unadjusted means ± SE somatic repeats as a function of age in *Hdh(Q150/Q150)/ogg1(+/+)* and *Hdh(Q150/Q150)/ogg1(-/-)* animals

^*c*.^ *Hdh(Q150/Q150)/ogg1(+/+)* and *Hdh(Q150/wt)/ogg1(+/+)* animals

^*d*.^ *Hdh(Q150/Q150)/ogg1(-/-) and Hdh(Q150/wt)/ogg1(-/-)* animals

^*e*.^ *Hdh(Q150/Q150)/ogg1(+/+)* animals

^*f*.^ *Hdh(Q150/Q150)/ogg1(-/-)* animals

### Motor function depends on both the HD and the OGG1 genotypes in an opposite manner

**Fig 4:**
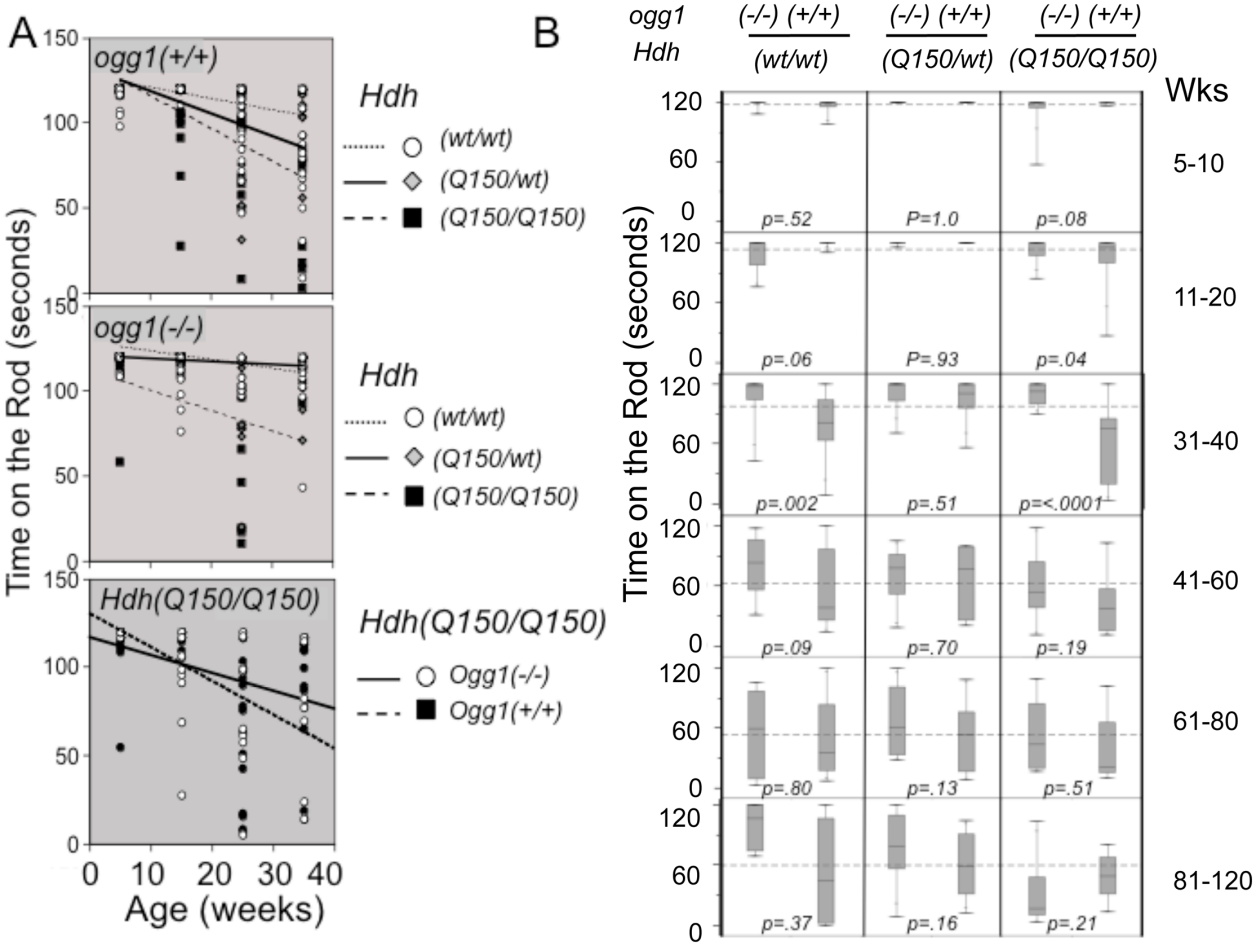
Suppression of somatic expansion delays motor decline in animals with similar inherited repeats. (A) The trends of motor decline versus genotype, as judged by linear fits of the averages. Motor performance in animals is affected by the m*HTT* and *OGG1* genotype in an opposite manner. *(top panel)* Motor decline depends on the complement of mHTT in the presence of OGG1. *(middle panel)* Loss of two *OGG1* alleles suppresses the average motor decline in *Hdh(Q150/150)/ogg1(-/-)* animals that express only one *mHTT* allele. (*lower panel*) Direct comparison of the motor decline in *Hdh(Q150/150)/ogg1(+/+)* and *Hdh(Q150/150)/ogg1(-/-)* littermates. Points are plotted mid-range of the age group, ie. 15 weeks is representative of the whole 11–20 week age group. (B) Box and whisker plots for rotarod performance (time on the rod) versus *Hdh* and *ogg1* genotypes. The thin vertical lines (whiskers) represent the entire distribution of performances at the indicated ages (see S6B Fig). Boxes represent the median 50% of performance values, with 25% above the median and 25% below the median. The horizontal line in the box indicates the median. The dotted horizontal line across the plot in each age group is the global average of performances when all 6 genotypes are combined. N = 16–20 animals per age group per genotype. Motor function was measured using a rotarod apparatus with a fixed-speed protocol described previously [36, 49, 50].

We hypothesized that if somatic expansion contributed to toxicity, then we could make two predictions: (1) motor decline in *Hdh(Q150/Q150)/ogg1(-/-)* and *Hdh(Q150/wt)*/*ogg1(-/-)* should be delayed relative to their *Hdh(Q150/Q150)/ogg1(+/+)* and *Hdh(Q150/wt)*/*ogg1(+/+)* littermates, and (2) the delay should occur within the age range in which the somatic and inherited repeats were best resolved. Both predictions were supported by the results. The motor deficits were measured using two conventional tests [31]: maneuvering a rotating rod (rotarod) (Fig 4) and grip strength (S6 Fig).

The performance of animals in all genotypes was highly variable, consistent with the heterogeneous distribution of repeat sizes in each animal (~100 repeat spread) (Fig 2), and reminiscent of variability in human HD patients (S1A Fig) [14]. Nonetheless, we found clear trends in motor performance among genotypes (Fig 4A), although the average performance times did not achieve statistical significance in simple linear fits (Fig 4A). While the number of mutant alleles did not affect the size of the somatic expansions, motor decline was greater in animals expressing two disease-length alleles (expressing twice the mutant protein), and occurred at a younger age (Fig 4A, panel 2). Homozygous *Hdh(Q150/Q150)/ogg1(+/+)* animals performed markedly worse in the first 40 weeks of life relative to heterozygous *Hdh(Q150/wt)/ogg1(+/+)* or *Hdh(wt/wt)* animals, and loss of OGG1 improved the average performance (Fig 4A, panels 2 and 3). In the absence of OGG1, motor decline was greater in animals expressing two disease-length alleles and occurred at a younger age (Fig 4A, panel 2), and was not substantially different in this late onset model from *Hdh(wt/wt)* littermates within the first 40 weeks. The same trends were observed when animals were tested by grip strength (S6 Fig). Motor decline in *Hdh(Q150/wt)/ogg1(+/+)* animals expressing only one allele occurred later, and as previously noted in this line (Lin et al., 2007), was not substantially different in this late onset model from *Hdh(wt/wt)* littermates within the first 40 weeks.

The variability across a distribution becomes a robust statistical parameter using a Tukey quartile-based approach [32]. Indeed, when the entire distribution of performances was considered, it was obvious that loss of OGG1 suppressed motor decline (Fig 4B). In the box and whisker plots (schematically explained in S6B Fig), the length of the thin line for each genotype (the whisker) visualizes the entire range of performance values from shortest time on the rod to longest time on the rod for each genotype (Fig 4B). The performances were divided into quartiles around the median value; the boxes represent the median 50% of the performances; 25% above and 25% below the median (S6B Fig). The whiskers above and below the box are the best and worst 25% performances, respectively.

The majority of the *Hdh(Q150/Q150)/ogg1(-/-)* mice significantly outperformed their *Hdh(Q150/Q150)/ogg1(+/+)* littermates (Fig 4B). Between 5–10 weeks, greater than 75% of their performances from all genotypes overlapped, but by 11 weeks, significant differences emerged. The performances of the *Hdh(Q150/Q150)/ogg1(+/+)* mice were poorer relative to any other genotype of comparable age, and remained so up to 40 weeks (Fig 4B, 11–20 weeks). With respect to OGG1, the median performance time of the *Hdh(Q150/Q150)/ogg1(+/+)* mice and *Hdh(Q150/Q150)/ogg1(-/-)* during this period was 119 seconds and 117 seconds, respectively, but roughly 75% of the performances of the *Hdh(Q150/Q150)/ogg1(-/-)* animals were better than those of their *Hdh(Q150/Q150)/ogg1(+/+)* littermates (Fig 4B). Indeed, the median 50% of performances for the latter ranged between 120 and 85 seconds (Fig 4B, the boxes), and more than half of them overlapped with the lowest quartile of their *Hdh(Q150/Q150)/ogg1(-/-)* littermates. The poorest quartile of *Hdh(Q150/Q150)/ogg1(+/+)* performances were as low as 25 seconds (Fig 4B), as compared to the poorest quartile of the *Hdh(Q150/Q150)/ogg1(-/-)* performances, which did not fall below 75 seconds (Fig 4B). Thus, loss of OGG1 across the entire distribution led to significant improvement around 11–20 weeks.

At 31–40 weeks, the improvement was even clearer. The median performances of the *Hdh(Q150/Q150)/ogg1(+/+)* and *Hdh(Q150/Q150)/ogg1(-/-)* littermates were different, 118 and 78 seconds, respectively. The entire performance distribution of the *Hdh(Q150/Q150)/ogg1(-/-)* fell close to the median (between 118 and 90 seconds), and was similar to their performance of 11 weeks (Fig 4B, the box for *Hdh(Q150/Q150)/ogg1(-/-)*). In contrast, the median 50% of performances of the *Hdh(Q150/wt)/ogg1(+/+)* animals did not overlap with the worst performance times of their *Hdh(Q150/Q150)/ogg1(-/-)* littermates (Fig 4B, 31 weeks). Indeed, the performance of *Hdh(Q150/wt)/ogg1(-/-)* animals at that age was similar to that of their wild-type counterparts, and, loss of OGG1 modestly offset the effects of expressing the two mutant alleles (Fig 4B, compare *Hdh(Q150/Q150)/ogg1(-/-) and HdhQ(wt/wt)/ogg1(-/-))*. The onset of motor decline in the *Hdh(Q150/Q150)/ogg1(+/+)* (11–20 weeks), and *Hdh(Q150/Q150)/ogg1(-/-)* littermates (41–50 weeks) differed minimally by 30 weeks and maximally by 40 weeks. Thus, loss of OGG1 in the homozygous *Hdh(Q150/Q150)/ogg1(-/-)* crosses led to a remarkable 7–10 month delay in the onset of motor decline relative to *Hdh(Q150/Q150)/ogg1(+/+)* littermates, who had inherited the same disease-length allele. Motor decline in *Hdh(Q150/Q150)/ogg1(+/+)* animals was obvious by 11 weeks, reached a maximum around 31 weeks and was not different among genotypes past 40 weeks. Whether analyzed by quantile analysis, linear regression, or by the average, loss of OGG1 in the *Hdh(Q150/Q150)/ogg1(-/-)* crosses conferred a substantial improvement on motor function within the period predicted by the somatic expansion window.

Linear regression described statistically significant relationships [32]. When all animals were combined, the average time on the rod was 91.9s; females performed 7% better (an improvement of 7.7s), loss of OGG1 improved performance by 12% (11s), and harboring the two HD alleles reduced performance by 26% (23.7 seconds) (S5B Fig). These striking findings provided evidence that somatic expansion contributed to pathophysiology, and suppression of somatic expansion was beneficial. Since the only known effect of OGG1 on DNA is its repair function, and *Hdh(Q150/Q150)/ogg1(+/+)* and *Hdh(Q150/Q150)/ogg1(-/-)* animals were indistinguishable by other measures, reduction in the somatic expansion appeared to drive the motor improvement.

### Somatic expansion influences the onset of disease

Although previous measures predicted relationships in postmortem brain, our measurements provided a means to determine a quantitative relationship between phenotype and somatic length at the time of onset. Linear regression and quantile analysis determined whether the size of the somatic expansion in these animals aligned with “good” and “bad” performance on the rotarod (Table 2). The pooled motor performance over 40 weeks was adjusted for age, gender, HD and OGG1 status for each quantile of the CAG repeat distribution. At the time of onset, the repeats at the lower end of the distribution (smaller repeats) significantly associated with better motor performance, consistent with earlier extrapolations from human postmortem brain [12], and all brain regions contributed equally to toxicity as judged by linear regression. In agreement with others [11–13], we observed that the striatum had the longest tract sizes. However, the predictive significance between performance and the repeat length at the upper extreme was statistically significant only in the hippocampus and the cerebellum (Table 2). As judged by quantile analysis (Fig 3), poor motor performance, when measured at the time of onset, correlated best with a larger number of smaller expansions that occurred across the entire distribution (Fig 3), rather than to the longest alleles (Table 2).

**Table 2:** The relationship between motor performance^a^ and the CAG repeats length in somatic tissues^b^.

Percentile of CAG repeats	Striatum^c^	Hippocampus^c^	Cerebellum^c^	Cortex^c^
1	P = 0.002*	P = 0.01*	P<0.0001*	P = 0.005*
5	P = 0.001*	P = 0.05*	P = 0.0006*	P = 0.05*
10	P = 0.005*	P = 0.04*	P = 0.001*	P = 0.11
20	P = 0.02*	P = 0.12	P = 0.006*	P = 0.21
30	P = 0.06	P = 0.23	P = 0.01*	P = 0.40
40	P = 0.07	P = 0.43	P = 0.03*	P = 0.54
50	P = 0.12	P = 0.42	P = 0.18	P = 0.66
60	P = 0.16	P = 0.62	P = 0.78	P = 0.74
70	P = 0.45	P = 1.00	P = 0.65	P = 0.85
80	P = 0.75	P = 0.62	P = 0.21	P = 0.85
90	P = 0.89	P = 0.08	P = 0.07	P = 1.00
95	P = 0.47	P = 0.04†	P = 0.01†	P = 0.69
99	P = 0.44	P = 0.008†	P = 0.01†	P = 0.86

* Less repeats associated with longer time on the rod

^†^ More repeats associated with shorter time on the rod

^a.^ Time on the rotarod at 20rpm

^b.^ All the changes in CAG length were pooled for each tissue from birth to 40 weeks, the time frame of onset.

^c.^ The significance of the association between motor performance and somatic expansion was determined by linear regression. The coefficients were adjusted for *Hdh(Q150)* genotype, *ogg1* genotype, age, and gender. P = probability.

Somatic expansion at or above 40 weeks lost its dependence on OGG1, and we could no longer assign the phenotypes exclusively to somatic expansion (Fig 4B). At these older ages, the repeat tract changes in *Hdh(Q150/Q150)/ogg1(-/-)* animals often equaled or exceeded those of their *Hdh(Q150/Q150)/ogg1(+/+)* counterparts. This is most likely due to the action of other glycosylases or nucleotide excision repair enzymes that back-up OGG1 in removing oxidative DNA damage as the number of lesions rises [33–35]. The decline in motor function mirrored these changes, and we observed little difference among genotypes at older ages (Fig 4B). By histological examination (S7A Fig), the number of neurons declined and inclusions became apparent in both *Hdh(Q150/Q150)/ogg1(-/-)* animals and *Hdh(Q150/Q150)/ogg1(+/+)*, compared to *Hdh(wt/wt)*, animals between 50 and 100 weeks (S7A Fig). Thus, somatic expansion continued above 40 weeks, but the distinction between the two genotypes and the effects of the inherited and somatic expansions were unresolved (S7B Fig).

### Therapeutic suppression of somatic expansion accompanies the delay of onset and progression of HD phenotypes in mice

**Fig 5:**
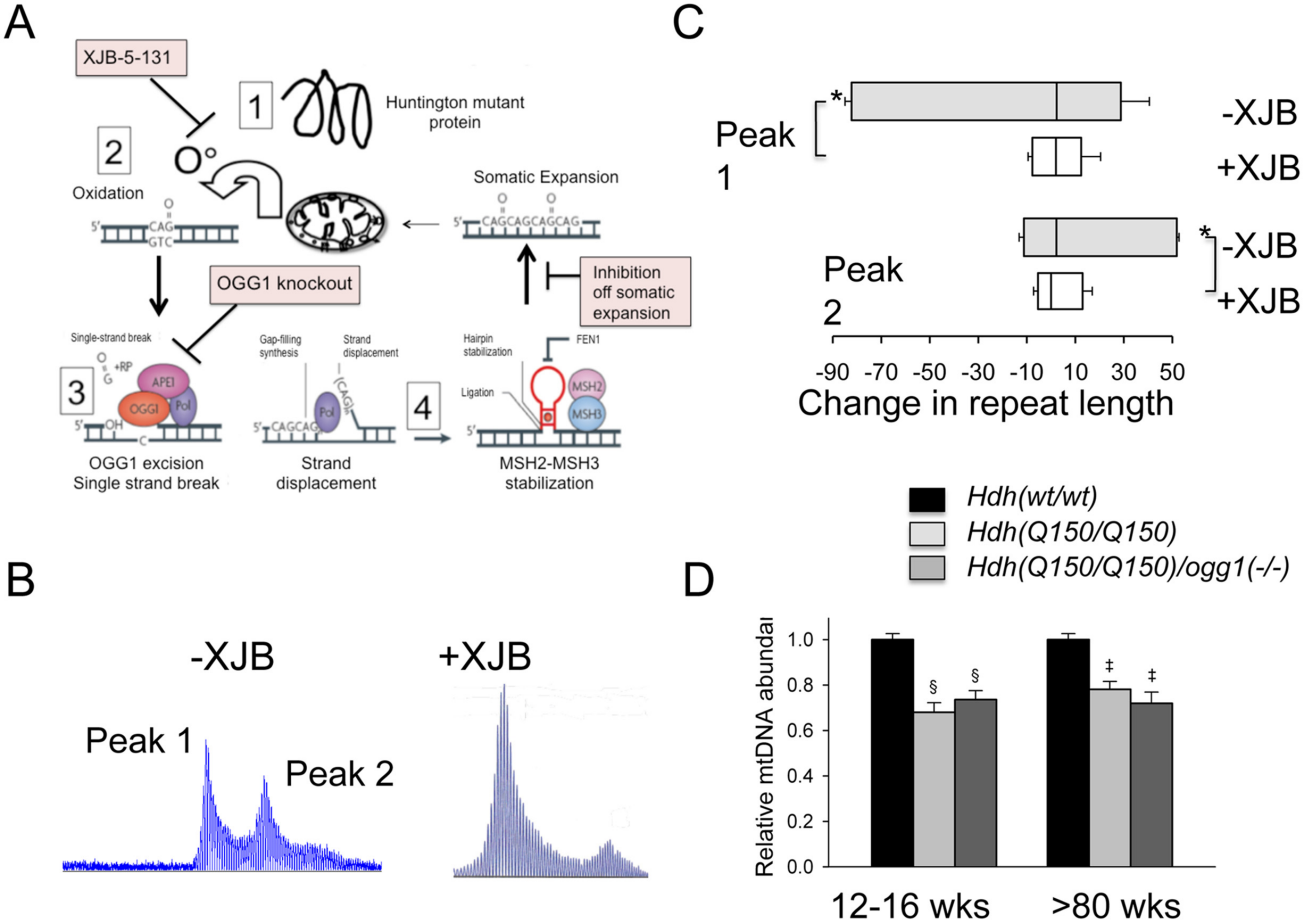
Pharmacological intervention suppresses oxidative DNA damage and somatic mutation *in vivo*. XJB-5-131 synthesis [51] and administration is as previously described [36]. (A) OGG1 and XJB-5-131 act in the same expansion pathway. Schematic diagram for the mechanism of somatic expansion (adapted from [1]), and the point of inhibition by XJB-5-131 or loss of OGG1. XJB-5-131 reduces oxidative DNA damage (the substrate for OGG1) and loss of OGG1 reduces base excision and single strand break intermediates for expansion by BER. (B) Representative examples of GeneScan analysis of *Hdh(Q150/Q150)/ogg1(+/+)* striatum of animals untreated or treated with XJB-5-131. (C) Quantification of repeat changes ± standard deviation for animals age 21–30 weeks. The somatic expansions in XJB-5-131-treated *Hdh(Q150/Q150)/ogg1(+/+)* animals are smaller than in untreated animals in the striatum. N = 6 per group (with and without XJB-5-131 treatment) *p<0.001. (D) Levels of mtDNA abundance in cerebral cortex of *Hdh*(*wt/wt)*, *Hdh(Q150/Q150)/ogg1(+/+)* and *Hdh(Q150/Q150)/ogg1(-/-)* animals at 12–16 weeks (n = 6) and >80 weeks of age (n = 6–9). ^§^p<0.0001 versus 12–16 weeks WT and ^‡^p<0.0001 versus >80 weeks WT.

Since expansion occurs in the process of OGG1-removal of oxidized bases, we hypothesized that lowering the number of oxidative lesions would reduce somatic expansion in *Hdh(Q150/Q150)/ogg1(+/+)* animals (Fig 5A). We have previously reported that pharmacological treatment with XJB-5-131, a mitochondrial-targeted scavenger of reactive oxygen species (ROS), reduces oxidative damage and breaks in mitochondrial DNA *in vitro*, and prevents motor decline in *Hdh(Q150/Q150)/ogg1(+/+)* animals *in vivo* [36](S8 Fig). We collected tissue from these animals [36], and tested whether suppression of somatic expansion in these animals accompanied the improvement in motor function [36] (Fig 5). Indeed, pharmacological treatment with XJB-5-131 not only suppressed motor decline (S8 Fig), but also inhibited somatic expansion of *Hdh(Q150/Q150)/ogg1(+/+)* in these animals at all ages tested relative to untreated animals (Fig 5B and 5C).

XJB-5-131 reduces the oxidative DNA substrates for OGG1, and we predicted that the compound would act in the same pathway as OGG1 to reduce somatic expansion (Fig 5A). Since XJB-5-131 suppressed mitochondrial damage during disease progression, we tested whether somatic expansion, mitochondrial function, or both correlated with the improvement in motor function in *Hdh(Q150/Q150)/ogg1(-/-)* mice (Fig 5D and S8 Fig). Little suppression was observed in the mitochondrial copy number from *Hdh(Q150/Q150)/ogg1(-/-)* mice below 80 weeks (Fig 5D). There was a reduction in copy number at 15 and 80 weeks in *Hdh(Q150/Q150)/ogg1(+/+)* compared to wild-type animals, consistent with the reported alteration in mitochondrial biogenesis [37]. However, the decrease in copy number was indistinguishable from that in *Hdh(Q150/Q150)/ogg1(-/-)* littermates, implying that somatic expansion contributed to the improvement in motor performance.

## Discussion

Here we report, for the first time, that somatic expansion contributes to Huntington’s disease toxicity. Loss of somatic expansion in the *Hdh(Q150/Q150)/ogg1(-/-)* crosses delays the onset of disease by around 7–10 months relative to their *Hdh(Q150/Q150)/ogg1(+/+)* littermates, although they both inherit a similar disease-length HD allele. The suppression of somatic growth is not strain dependent. We have previously reported that loss of OGG1 also suppresses somatic expansion in the *R6/1/ogg1(-/-)* animals [19]. Indeed, based on the average lengths, 70% of the latter animals displayed suppression of somatic expansion [19] relative to control animals.

The remarkable delay in motor decline is also not explained by differences in genetic background. The *Hdh(Q150/wt)* [20] and *ogg1(+/-)* [21] animals were extensively backcrossed over a five-year period to generate isogenic strains. There is no overt phenotype conferred by loss of OGG1 at disease onset. Thus, the beneficial effects observed in *Hdh(Q150/Q150)/ogg1(-/-)* animals appear to arise from reduction of somatic expansion. A shift of 7–10 months in the mouse translates to roughly 25 years in humans. Minimally, we predict that a shift in pathological onset of this magnitude is likely to make a difference in the quality of life of an HD patient.

Loss of NEIL1 [33], Cockayne’s syndrome-B (CSB) [35] and XPA [34] in mice reduces expansion, bolstering the idea that removal of oxidative DNA damage causes instability. However, the effects on pathophysiology in these animals are unknown. Loss of mismatch repair also attenuates expansion [38–45], but at the same time leads to methylation tolerance, hyper-recombination, tumors, lymphomas at early ages (peak at 8 weeks of age) [46], as well as global instability in repetitive elements throughout the genome [47, 48]. Linking the onset of pathophysiology to expansion in the *HTT* locus has not been possible. In contrast, the OGG1 knockout inhibits expansion and is advantageous in its lack of overt toxicity during the observation period. Consistent with the mouse genetic experiments, we report here that treatment with a pharmacological inhibitor, XJB-5-131, shortens and/or prevents lengthening of the repeat tracks during life (here), and rescues motor decline in these animals (S8 Fig). The results provide evidence that pharmacological approaches to offset disease progression are possible.

**Fig 6:**
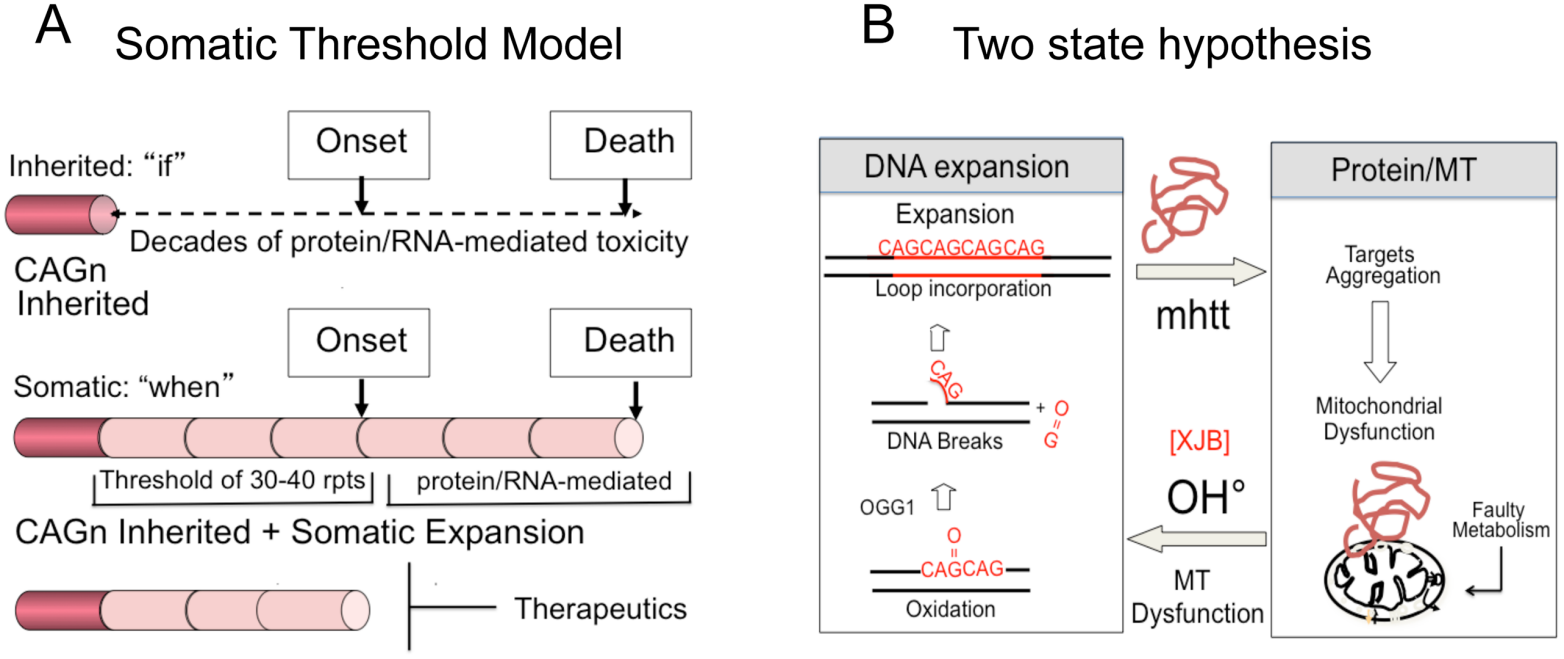
Model for somatic expansion and the age of disease onset. (A) In a conventional model, expansion arises from decades long toxic effects of expanded protein or RNA. Intervention is limited to breaking mHTT interactions with cellular proteins, which has not yet been therapeutically effective. In a somatic threshold model, toxicity arises when an inherited allele reaches a somatic length that is sufficient to support sustained toxicity. (B) We propose a two-state model for toxicity. The inherited repeats govern “if” disease will arise, while the somatic expansion governs, at least in part, the “when”. Intervention is possible by blocking the somatic expansion and delaying onset of disease.

Inhibition of somatic expansion, thereby, changes the therapeutic landscape. It has never been clear why obvious phenotypic onset in HD patients does not occur for decades, while the mutation is present from conception. We propose that the onset of toxicity is the sum of the inherited and somatic expansions. The latter provides a temporal bridge between inheriting the disease-length allele and the onset of disease (Fig 6A). In a conventional model, the onset of disease depends on the length of the inherited allele (Fig 6B and S1A). Disease potential is determined at birth and arises from the decades-long toxic effects of a mutant protein or RNA (Fig 6B). Therapeutics, in this case, is limited to inhibiting the effects of toxic protein-protein or RNA-protein interactions, which has not yet been successful.

A contribution of somatic expansion, however, implies that the inherited repeat does not entirely govern onset, which would be shifted by the size of the somatic changes that occur during life. In a somatic threshold model, onset arises when a somatic expansion produces a protein and/or RNA of sufficient length to sustain toxicity (Fig 6B). In such a model, the inherited CAG repeat length determines “if” disease will occur, but somatic mutation accounts for, at least in part, the “when” (Fig 6B). Suppressing somatic expansion delays disease onset. Our results provide hope that intervention for expansion diseases is possible, despite inheriting a dominant disease allele, and widens the therapeutic window for more than a dozen fatal diseases.

## Materials and Methods

### Animals and breeding

The Institutional Animal Care and Use Committee approved all procedures. Animals were treated under guidelines of ethical treatment of animals, and approved by IACUC protocol #274005 at Lawrence Berkeley Laboratory. All animal work was conducted according to relevant national and international guidelines.

The following mouse models were used: homozygous *Hdh(Q150/150)* and heterozygous *Hdh(Q150/wt)* (Lin et al., 2001) and ogg1 null mice (Klungland et al., 1999), which have been previously described and characterized. The *Hdh(Q150/wt)* and *ogg1(-/-)* mice were generated on the C57BL6 background. Therefore, we used *C57BL6* (Cnt) mice as controls, referred to as *Hdh(wt/wt)*. Heterozygous *Hdh(Q150/wt)* (Lin et al., 2001) and ogg1(+/-) mice (Klungland et al., 1999) were bred on C57BL6 background to obtain *Hdh(Q150/Q150)* and *ogg1(-/-)* mice. Each line was characterized by histology and for the level of expressed protein. Genescan analysis, as described previously, determined the size of the CAG repeat tracts as a function of age and brain region (Kovtun et al., 2007). Data were analyzed using GeneMapper software v4. Genescan analysis of the XJB-5-131 treated tissue was performed at LBNL.

### Antibodies, immunofluorescence and western analysis

Primary antibodies were: mouse OGG1 (1:1000, a kind gift from Tapas Hazra at University of Texas Medical Branch), mouse monoclonal Huntingtin (HTT) (1:1000, MAB2170, EMD Millipore, MA), and actin-HRP conjugated (1:5000, sc-1616, Santa Cruz Biotechnology). Tissue extracts were prepared in NP-40 lysis buffer (50 mM Tris-HCl pH 8.0, 150 mM NaCl, 1% Igepal Ca-630 and protease inhibitors (Complete, Roche). Tissue was washed twice with ice- cold PBS and resuspended in NP-40 lysis buffer, and kept on ice for 30 min. Then, the cellular suspension was centrifuged at 21,000xg for 5 min and the protein concentration in the supernatant was determined with BioRad DC Protein Assay Kit using albumin as a standard. Twenty five-fifty microgram of protein was separated using 10% SDS-PAGE. Anti-mouse HRP linked secondary antibody (1:1000, #7076S, Cell Signaling) was used and membrane was visualized with Pierce ECL Western Blotting Substrate (#32106, Thermo Scientific) using G:BOX with GeneSnap software form SynGene.

### Morphology

At least three mice from all nine genotypes and ages were taken into analysis. Mice were decapitated with a guillotine and the brains isolated. Brain hemispheres were post-fixed for at least 24 hours in buffered, 4% PFA. Paraffin- embedded, 4-μm-thick coronal sections were stained using a BondMax™ (Leica Microsystems GmbH/Menarini, Germany) automated immunostaining system. Analysis was conducted on 5–10 sections per mouse. Sections were pretreated with Citrate, EDTA or Enzyme 1 pretreatment solutions (Menarini, Germany) and immunostained using anti-IBA1 (EDTA pretreatment 20 min, 1:1,000 for 15 min, Wako GmbH, Germany), anti-GFAP (Enzyme 1 pretreatment, 1:500 for 15 min, DAKO, Germany), anti-NeuN (clone A60, Citrate pretreatment 20min, 1:500 for 15 min, Chemicon, Germany), anti-Ubiquitin (clone Ubi-1, EDTA pretreatment 20 min, 1:10,000 for 15 min, Millipore, Germany) and the Bond™ Polymer Refine Detection kit (Menarini, Germany) as described in (Scheffler et al., 2012). Whole tissue sections were fully digitized at a resolution of 230nm using a Mirax Midi slide scanner (Zeiss, Germany) as described in (Krohn et al., 2011) and 10 fields of view (FOV) at a natural magnification (1:1, 230nm per pixel, 53,3 fold on a 24” screen) were analyzed semi-automatically using the BX Analysis software package and a custom programmed macro (Keyence, Germany).

### Motor testing

Motor testing encompassed both rotarod and grip test, as described (Trushina et al., 2014; Xun et al., 2012). Weight and littersize were also quantified. Animals in each group were evaluated for rotarod performance and grip strength at the indicated ages. Mice were lowered onto the already spinning Rota-Rod (Ugo Basile) at the required speed (10 and 20 rpm were used in this study). The amount of time the animals stayed on the Rota-Rod was determined by a built-in magnetic trip-switch, which was stopped when the animal fell off. Mice were timed on the Rota-Rod for a maximum of 120s, with three attempts given for each mouse to attain 120s. Animals were tested for one session each day at each speed, for 5 consecutive days, and the best times for each trial were averaged for each animal. For grip strength test, mice were lowered onto a parallel rod (D < 0.25 cm) placed 50 cm above a padded surface. The mice were allowed to grab the rod with their forelimbs, after which they were released and scored for their success in holding onto the bar for 30 s. Mice were allowed three attempts to pass the bar test each day of testing, and were tested for 5 consecutive days. Any one successful attempt to hold onto the bar was scored as a pass. The percentage of animals that fell (and failed the test) was measured and recorded as a percentage of the total number of animals tested per genotype and age group. Mice were immediately sacrificed at the end of the 5-day testing period. Average number of mice tested per genotype and age group was greater than 12, with an approximately equal male:female ratio (407 males:351 females) in the 6 genotypes that were focused on for analysis.

### DNA extraction and CAG PCR amplification

DNA was prepared from mouse brain tissues and tails at the age indicated using the MasterPure Complete DNA and RNA Purification Kit (Epicentre Biotechnologies). Samples were incubated with Proteinase K, RNase A treated, followed by protein precipitation and centrifugation to remove cellular debris. DNA was precipitated with isopropanol, washed and resuspended in H2O. Amplification of CAG repeats from Hdh(Q150/wt)/ogg1(+/+), Hdh(Q150/Q150)/ogg1(+/+), Hdh(Q150/wt)/ogg1(-/-), and Hdh(Q150/Q150)/ogg1(-/-) mouse DNA was performed with a HEX-labeled forward primer (CCCATTCATTGCCTTGCTG) and reverse primer (GCGGCTGAGGGGGTTGA) in 15 μl reactions containing 0.2 mM dNTPs, 2 M betaine, AM buffer (67 mM Tris·HCl, pH 8.8/ 16.6 mM (NH4)SO4/ 2 mM MgCl2/ 0.17 mg/ml BSA) and 1 unit of Kapa Taq HotStart DNA Polymerase (Kapa Biosystems). Cycling conditions were as follows; 5 min at 94°C; 30 s at 94°C, 30 s at 60°C, 3 min at 72oC for 5 cycles; 30 s at 94°C, 30 s at 55°C, 5 min at 72°C for 38 cycles; 5 min at 72°C. Genescan analysis was performed using GeneMapper software v4. The statistical program R was used to separate partially overlapping curves in homozygous animals.

### Peak quantification

There are three groups. The initial allele distribution of the inherited repeat is subtracted from the somatic repeats at the age of interest to normalize changes. R uses an iterative curve fitting routine to a Gaussian simple peak shape model. The heterozygous (HdhQ150/wt) animals have only one peak to fit. For homozygous animals (HdhQ150/Q150), if the two peak distributions are coincident or are very far apart, the initial allele distribution of the inherited repeat is the same as for heterozygous animals. If the peaks are partially overlapping, we use iterative fitting routines of R (the statistical program) to resolve them. Mathematical resolution of the two peaks occurs only once (i.e., we do not follow the same animals with time and compound errors by re-fitting the results from the same animals at multiple ages). In our case, we fit to a Gaussian function using two non-linear parameters: peak position and peak width (the peak height is a linear parameter and is determined by regression). In R, peak resolution is not performed by linear least-squares methods because such signals cannot be modeled as polynomials with linear coefficients (the positions and widths of the peaks are not linear functions). Compared to the simpler polynomial least-squares methods for measuring peaks, the iterative method has the advantage of using all the data points across the entire peak, including zero and negative points. This method can be applied to resolve multiple overlapping peaks to a high degree of accuracy.

### Least squares regression

Least squares regression analysis (Cohen et al., 1993) was used to compare genotype and motor performance. The ogg1(-/-) and Hdh(Q150/Q150) genotypes could affect overall performance (represented by different intercepts). The ogg1(-/-) and Hdh(Q150/Q150) genotypes could also interact in affecting performance, and potentially include six separate intercepts: six separate age effects, and all their interactions, in addition to covariates. To simplify the model, we included separate intercepts for each genotype in a model that included sex and separate age effects for each Hdh(Q150/Q150) genotype. This allowed us to combine certain genotypic-specific intercepts and age effects into a simpler form that included three intercepts and a regression slope for age. Values were expressed as mean ± standard error of the mean (SEM), unless otherwise stated. P-values were obtained from the unpaired two-tailed Student's t-test.

Statistical analyses of means for three or more groups were performed using one-way analysis of variance (ANOVA) with the categories of genotype and age as independent factors followed by the Newman-Keuls post-hoc test for multiple comparison. For analyses of means involving only two groups with a sample size n<30, the F-test was used to determine whether the variances between the two groups were significantly different. For samples with a significant difference in variance, the Welch’s t-test was applied. Student’s t-test was applied for the samples (n ≥ 30) with an insignificant difference in variance. The significance level was set at 0.05 for all analyses. All statistical computations were carried out using Prism (Graphpad Software).

### XJB-5-131 synthesis and treatment

Treatment using XJB-5-131 and the motor testing results are previously described (Xun et al., 2012). The tissue used for sizing of the somatic repeats length was obtained from the same animals whose motor performance was reported (Xun et al., 2012). XJB-5-131 was synthesized as described previously (Wipf et al., 2005). Hdh(Q150/Q150)/ogg1(+/+) mice were intraperitoneally injected with 1 mg/kg of XJB-5-131 or phosphate buffered saline three times per week from 7 to 57 weeks. At least seven animals were tested in each age group per genotype.

### Analysis of mtDNA abundance by quantitative PCR

The relative level of mtDNA abundance in mouse cerebral cortex was performed as previously described (Ayala- Torres et al., 2000; Siddiqui et al., 2012). The determination of mtDNA abundance consisted of amplifying a 116 bp mtDNA fragment by performing an initial denaturation for 45 s at 94°C, followed by 22 cycles of denaturation for 15 s at 94°C, annealing/extension at 61°C for 45 s, and a final extension for 45 s at 72°C. We used the following primer nucleotide sequences: 5′-CCCAGCTACTACCATCATTCAAGT-3′ (forward) and 5′-GATGGTTTGGGAGATTGGTTGATGT-3′ (reverse). The relative copy numbers were calculated as the relative amplification of the Hdh(Q150/Q150)/ogg1(+/+) cortex or the Hdh(Q150/Q150)/ogg1(-/-) cortex compared to the wild-type Hdh(wt/wt)/ogg1(+/+) controls. The results were derived from two qPCR assays in duplicate on each animal. Six mice were used in each analysis.

## Supporting Information

S1 Fig Somatic expansion in humans and in mice. (A) Variability of onset with CAG repeat length in human HD patients. The red line indicates the more than 40 year spread in onset variability of an individual with the same repeats size (44 CAG repeats). Black points indicate onset range at the indicated age. Confidence limits are indicated at right. Figure modified from *Andrew S*.*E*. *et*. *al*. *Nature Genetics (1993)*, *4*, *1993*, *398–403*. (B) Distribution of repeat sizes of 11 month HD animals. Taken from *Kennedy*, *L*., *Evans*, *E*., *Chen*, *C*.*M*., *Craven*, *L*., *Detloff*, *P*.*J*., *Ennis*, *M*. *and Shelbourne*, *P*.*F*. *Dramatic tissue-specific mutation length increases are an early molecular event in Huntington disease pathogenesis*. *Human Molecular Genetics 12 (2001) p3359-67*. (C) Toxic oxidation cycle. mHTT induces cellular stress (Step 1) and enhances release of oxygen species from the mitochondria (Step 2). Somatic expansion arises in the process of repairing oxidative DNA damage. 7,8-dihydro-8-oxoguanine DNA glycosylase (OGG1) (red oval) recognizes and removes oxidized guanines (O = G) in the DNA template (Step 3). Removal of the oxidized guanine creates an apurinic site in which the widowed cytosine (C) has no partner. OGG1 can nick the phosphodiester backbone. The trinucleotide repeat (TNR) strand is displaced during gap-filling synthesis and TNRs from the displaced ‘flap’ can fold back into a hairpin (a flap containing CAG is shown as an example). Binding of the mismatch repair recognition complex MSH2–MSH3 (light pink and blue ovals) to the A-A mismatched bases (red circle in hairpin stem) stabilizes the hairpin (Step 4). The loop is not removed and becomes the precursor for expansion. The process repeats itself with age.
(TIF)

S2 Fig The breeding scheme for *Hdh(Q150)* (indicated here as *150* to save space) and *ogg1* genotypes. (A) The *HdhQ150* knock-in mice were generated in a *C57BL6* background. The OGG1 KO mice were generated by embryo injection into blastocysts from C57BL/6J mice. The *wt/wt* control *C57BL6* mice came from the breeding. In the crosses, each line is bred to maintain the heterozygous state until the last step when the homozygous strains are generated. The black arrow indicates that there may be breeding steps to amplify the number of heterozygous animals in a desired line for the final step of the homozygous state. The end step results in generation of all 9 genotypes. *Wt* arising from the breeding are used in the analysis. Breeding of the animal crosses started in 2007 to generate the isogenic lines. The red arrow indicates that populations of genotypes are stopped and aged for the designated number of weeks. All animals were aged, tested in the motor performance paradigm at a selected age, and immediately sacrificed for histology and CAG repeat analysis from brain tissue. (B) The litter size and weights at the indicated ages for all nine genotypes. Littersize for all nine genotypes was measured at birth.
(TIF)

S3 Fig Expression of OGG1 and HTT proteins in *Hdh(wt/wt) and Hdh(Q150/Q150)* animals. (A) Quantification (from Fig 1D) of age-dependence of OGG1 protein expression relative to actin in brain regions as indicated: Y is 7–10 weeks, M is 12–16 weeks; O is greater than 30 weeks. Values are plotted relative to OGG1 levels in young *Hdh(wt/wt)* (light grey) mice which are normalized to reference value of 1. (B) (WB:) Western Blot. The age-dependence of HTT/mHTT protein expression relative to actin controls, in cortex: Y is 7–10 weeks, M is 12–16 weeks; O is greater than 30 weeks.
(TIF)

S4 Fig The frequency of CAG tract length vs. the change in repeat tract size in *Hdh(Q150/Q150)* and *Hdh(Q150/wt)* mice at ages from 5–40 weeks in four brain regions. (A) Distributions for *Hdh(Q150/Q150)*; (B) Distributions for CAG tract length in *Hdh(Q150/wt)*. The HD genotype is indicated at the top of A and B. The *ogg1* genotype is indicated below the plots. The four regions of the brain are indicated.
(TIF)

S5 Fig CAG repeat length changes and motor performance. (A) The mean CAG repeat length changes at 10 weeks were 4.89±1.41 and 2.04±1.13 in *Hdh(Q150/Q150)/ogg1(+/+)* and *Hdh(Q150/Q150)/ogg1(-/-)* animals, respectively. The number of extreme expansions fell within +2σ and +3σ from the mean. Using Z-statistics, scores of 3 or larger indicate significant differences between any two groups. The Z scores of 4.50 indicated a strong suppression of somatic expansion in the striatum of *Hdh(Q150/Q150)/ogg1(-/-)* animals relative to *Hdh(Q150/Q150)/ogg1(+/+)* animals at 10 weeks. (B) Regression analyses of rotarod performance versus gender and genotype. Distribution of performance values expressed as “time on the rod”. Linear regression analysis of performance with the indicated variable.
(TIF)

S6 Fig Loss of OGG1 improved grip strength. (A) Animals of indicated age groups were allowed to grab with their forelimbs a narrow wire rod (D< 0.25 cm) suspended 50 cm above a padded surface. Each mouse was released and observed for 30 sec. Mice scoring positive for this test held onto the bar for at least 30 sec. The entire group of animals was tested together, and the results were expressed as a percent pass. *Hdh(Q150/Q150)/ogg1(+/+)* and *Hdh(Q150/Q150)/ogg1*(-/-) animals performed less well compared to controls (gray circles). The *Hdh(Q150/Q150)/ogg1*(-/-) animals most often out performed the *Hdh(Q150/Q150)/ogg1(+/+)* animals at comparable ages. In each mouse line, the percent of pass progressively decreased with age. By 40 weeks, about 62% of the *Hdh(Q150/Q150)/ogg1(+/+)* animals failed the test. In contrast, loss of OGG1 in *Hdh(Q150/Q150)/ogg1*(-/-) crosses conferred a substantial improvement on grip strength. (B) Hypothetical schematic of a box plot. (left) The entire distribution of performance values is indicated by the length of the thin line (from 10–115 seconds). The median is indicated by the horizontal black line in the box. The quartiles are indicated by the double arrows labeled 25%. (right) Fifty percent of values lie in the box: 25% above the median and 25% below the median. The most frequent 50% range is between 50–110 seconds, The whiskers above and below the box are the highest and lowest 25%, respectively.
(TIF)

S7 Fig Histological analysis of caudate-putamen. (A) Histology of the caudate/putamen of *Hdh(Q150/Q150)/ogg1(+/+)*, *Hdh(Q150/Q150)/ogg1(-/-)* and controls, *Hdh(wt/wt)/ogg1(+/+)* and *Hdh(wt/wt)/ogg1(-/-)* animals, around 50 weeks of age. H&E (Hematoxylin & Eosin stain), Luxol-Nissl (LN). The small black arrows indicate protein-rich inclusions. IBA1 (microgliosis marker), NeuN (neurons), and ubiquitin (Ubi), Black arrows indicate inclusions, as stated in text. Scale bar is 50μm except for Ubi staining which is 100μm. Genotypes are indicated. Quantification of neurons by NeuN staining comprised 3 animals, 5–10 tissues slices and 10 random fields on each slice. (B) One example showing scans in which expansion was larger in some tissues in whole brain in *Hdh(Q150/Q150)/ogg1(-/-) relative to Hdh(Q150/Q150)/ogg1(+/+)* and animals at 60 weeks. Expansion in both lines is similar. Examples of expansion distribution in individual mice in tail, brain, and liver, as indicated. Size markers are indicated in orange.
(TIF)

S8 Fig Somatic expansion in XJB-5-131-treated mice occurred concomitantly with the delay in onset observed in these animals. Tissue was taken from *Hdh(Q150/Q150)* animals reported by *Xun et al*., *2012*, and tested for expansion here (Fig 5). (A) Structure of XJB-5-131 and its fluorescent derivative BODIPY-FL-XJB-5-131. The tempol radical scavenger portion (red), and the MT targeting moiety gramicidin S (black) are indicated. (B and C) MT staining with BODIPY-FL-XJB-5-131 co-localizes in primary neurons with Mitotracker, a mitochondrial dye (MtT). (D) Suppression of motor decline by treatment. Time on the rod decreases in *Hdh(Q150/Q150) (here labelled as HD150KI)* animals and is restored in animals after 9, 28, and 52-weeks of treatment with XJB-5-131.
(TIF)
