# Supplementary figures and images for: Suppression of Somatic Expansion Delays the Onset of Pathophysiology in a Mouse Model of Huntington’s Disease
Source: PLoS Genet. 2015 Aug 6;11(8):e1005267. doi: 10.1371/journal.pgen.1005267 (PMC4527696; doi:10.1371/journal.pgen.1005267)

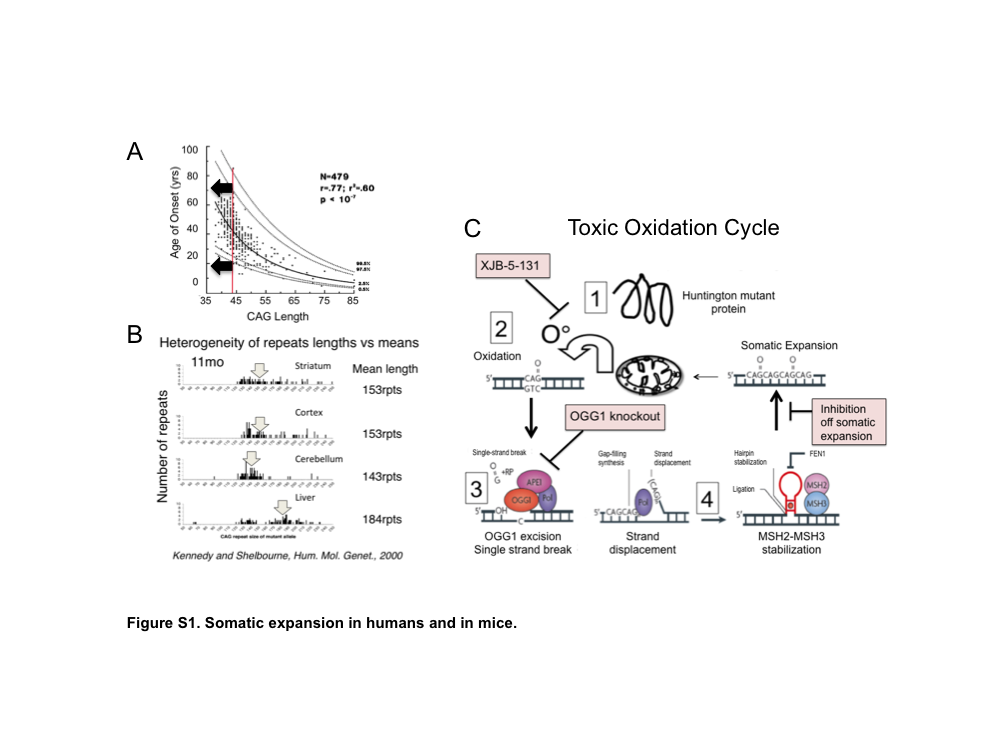

Supplement: S1 Fig — (A) Variability of onset with CAG repeat length in human HD patients. The red line indicates the more than 40 year spread in onset variability of an individual with the same repeats size (44 CAG repeats). Black points indicate onset range at the indicated age. Confidence limits are indicated at right. Figure modified from Andrew S.E. et. al. Nature Genetics (1993), 4, 1993, 398–403. (B) Distribution of repeat sizes of 11 month HD animals. Taken from Kennedy, L., Evans, E., Chen, C.M., Craven, L., Detloff, P.J., Ennis, M. and Shelbourne, P.F. Dramatic tissue-specific mutation length increases are an early molecular event in Huntington disease pathogenesis. Human Molecular Genetics 12 (2001) p3359-67. (C) Toxic oxidation cycle. mHTT induces cellular stress (Step 1) and enhances release of oxygen species from the mitochondria (Step 2). Somatic expansion arises in the process of repairing oxidative DNA damage. 7,8-dihydro-8-oxoguanine DNA glycosylase (OGG1) (red oval) recognizes and removes oxidized guanines (O = G) in the DNA template (Step 3). Removal of the oxidized guanine creates an apurinic site in which the widowed cytosine (C) has no partner. OGG1 can nick the phosphodiester backbone. The trinucleotide repeat (TNR) strand is displaced during gap-filling synthesis and TNRs from the displaced ‘flap’ can fold back into a hairpin (a flap containing CAG is shown as an example). Binding of the mismatch repair recognition complex MSH2–MSH3 (light pink and blue ovals) to the A-A mismatched bases (red circle in hairpin stem) stabilizes the hairpin (Step 4). The loop is not removed and becomes the precursor for expansion. The process repeats itself with age. (TIF) [file pgen.1005267.s001.tif]

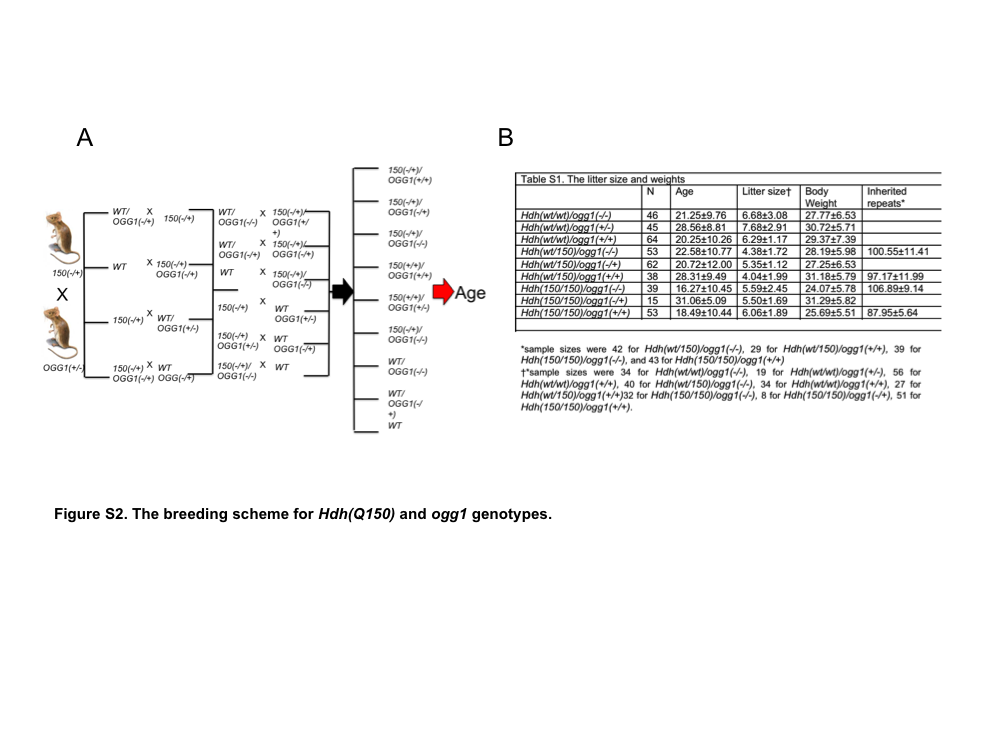

Supplement: S2 Fig — (A) The HdhQ150 knock-in mice were generated in a C57BL6 background. The OGG1 KO mice were generated by embryo injection into blastocysts from C57BL/6J mice. The wt/wt control C57BL6 mice came from the breeding. In the crosses, each line is bred to maintain the heterozygous state until the last step when the homozygous strains are generated. The black arrow indicates that there may be breeding steps to amplify the number of heterozygous animals in a desired line for the final step of the homozygous state. The end step results in generation of all 9 genotypes. Wt arising from the breeding are used in the analysis. Breeding of the animal crosses started in 2007 to generate the isogenic lines. The red arrow indicates that populations of genotypes are stopped and aged for the designated number of weeks. All animals were aged, tested in the motor performance paradigm at a selected age, and immediately sacrificed for histology and CAG repeat analysis from brain tissue. (B) The litter size and weights at the indicated ages for all nine genotypes. Littersize for all nine genotypes was measured at birth. (TIF) [file pgen.1005267.s002.tif]

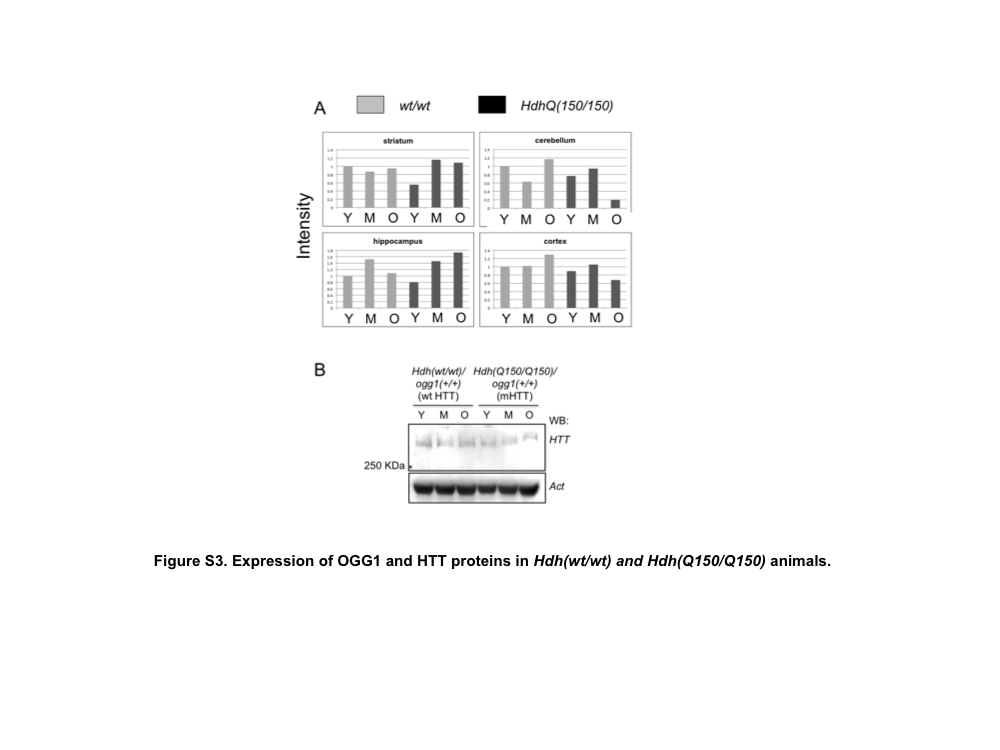

Supplement: S3 Fig — (A) Quantification (from Fig 1D) of age-dependence of OGG1 protein expression relative to actin in brain regions as indicated: Y is 7–10 weeks, M is 12–16 weeks; O is greater than 30 weeks. Values are plotted relative to OGG1 levels in young Hdh(wt/wt) (light grey) mice which are normalized to reference value of 1. (B) (WB:) Western Blot. The age-dependence of HTT/mHTT protein expression relative to actin controls, in cortex: Y is 7–10 weeks, M is 12–16 weeks; O is greater than 30 weeks. (TIF) [file pgen.1005267.s003.tif]

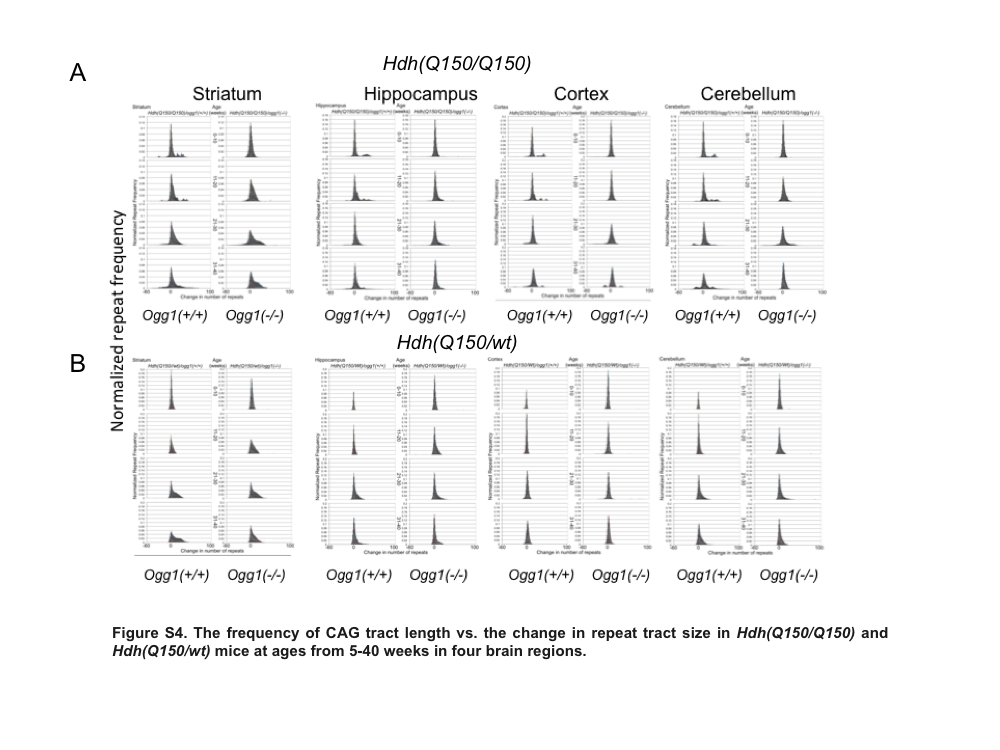

Supplement: S4 Fig — (A) Distributions for Hdh(Q150/Q150); (B) Distributions for CAG tract length in Hdh(Q150/wt). The HD genotype is indicated at the top of A and B. The ogg1 genotype is indicated below the plots. The four regions of the brain are indicated. (TIF) [file pgen.1005267.s004.tif]

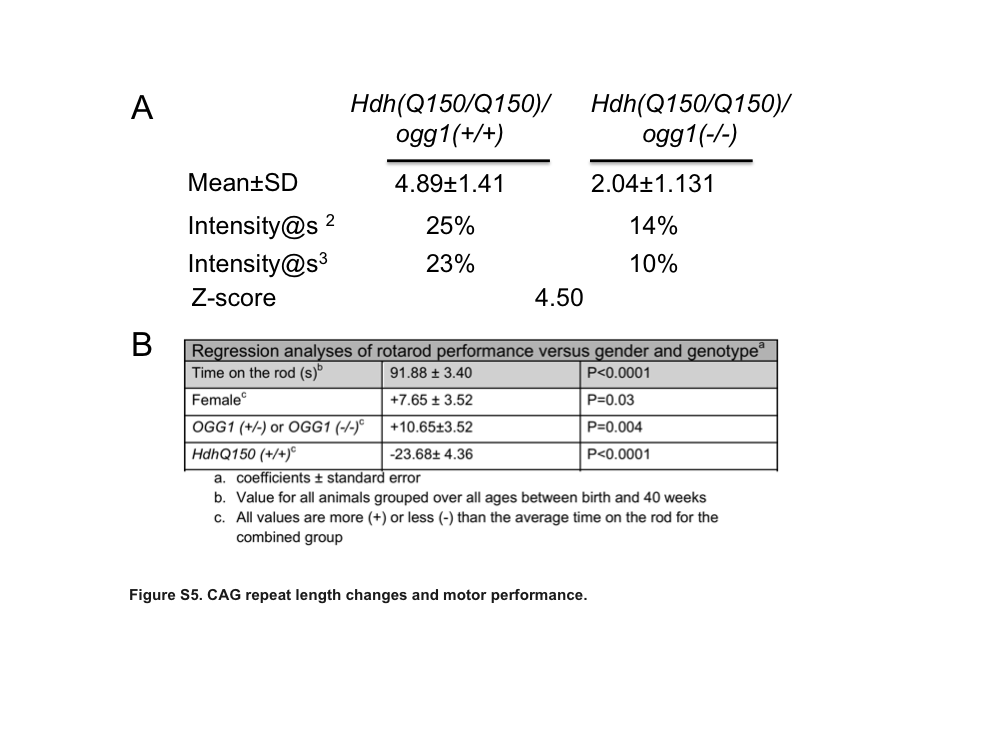

Supplement: S5 Fig — (A) The mean CAG repeat length changes at 10 weeks were 4.89±1.41 and 2.04±1.13 in Hdh(Q150/Q150)/ogg1(+/+) and Hdh(Q150/Q150)/ogg1(-/-) animals, respectively. The number of extreme expansions fell within +2σ and +3σ from the mean. Using Z-statistics, scores of 3 or larger indicate significant differences between any two groups. The Z scores of 4.50 indicated a strong suppression of somatic expansion in the striatum of Hdh(Q150/Q150)/ogg1(-/-) animals relative to Hdh(Q150/Q150)/ogg1(+/+) animals at 10 weeks. (B) Regression analyses of rotarod performance versus gender and genotype. Distribution of performance values expressed as “time on the rod”. Linear regression analysis of performance with the indicated variable. (TIF) [file pgen.1005267.s005.tif]

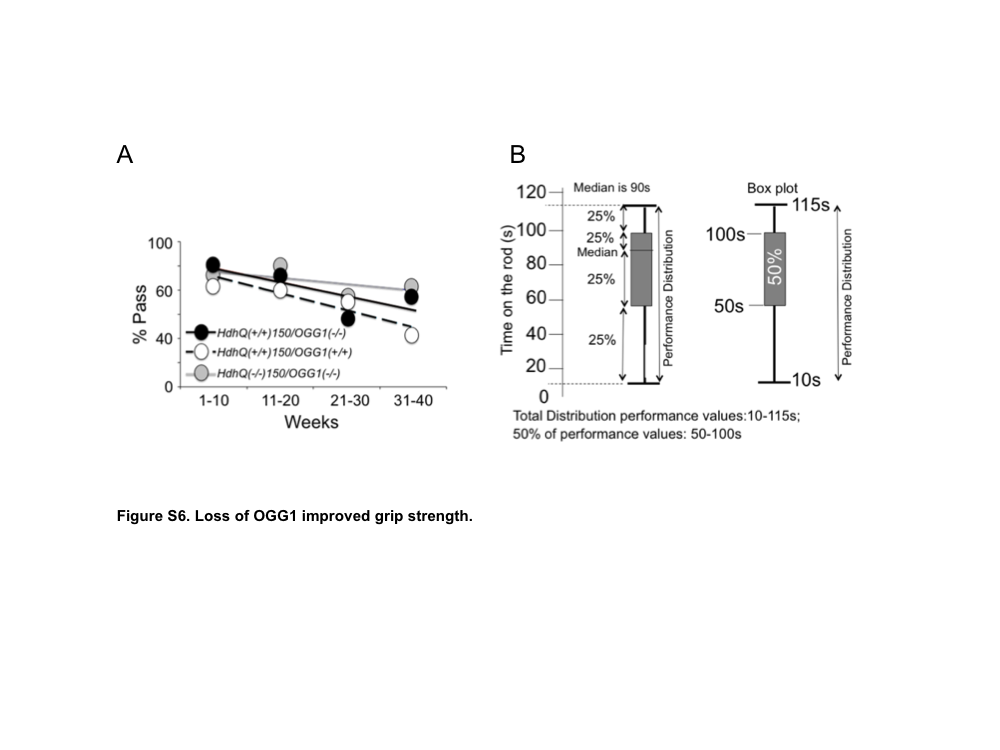

Supplement: S6 Fig — (A) Animals of indicated age groups were allowed to grab with their forelimbs a narrow wire rod (D< 0.25 cm) suspended 50 cm above a padded surface. Each mouse was released and observed for 30 sec. Mice scoring positive for this test held onto the bar for at least 30 sec. The entire group of animals was tested together, and the results were expressed as a percent pass. Hdh(Q150/Q150)/ogg1(+/+) and Hdh(Q150/Q150)/ogg1(-/-) animals performed less well compared to controls (gray circles). The Hdh(Q150/Q150)/ogg1(-/-) animals most often out performed the Hdh(Q150/Q150)/ogg1(+/+) animals at comparable ages. In each mouse line, the percent of pass progressively decreased with age. By 40 weeks, about 62% of the Hdh(Q150/Q150)/ogg1(+/+) animals failed the test. In contrast, loss of OGG1 in Hdh(Q150/Q150)/ogg1(-/-) crosses conferred a substantial improvement on grip strength. (B) Hypothetical schematic of a box plot. (left) The entire distribution of performance values is indicated by the length of the thin line (from 10–115 seconds). The median is indicated by the horizontal black line in the box. The quartiles are indicated by the double arrows labeled 25%. (right) Fifty percent of values lie in the box: 25% above the median and 25% below the median. The most frequent 50% range is between 50–110 seconds, The whiskers above and below the box are the highest and lowest 25%, respectively. (TIF) [file pgen.1005267.s006.tif]

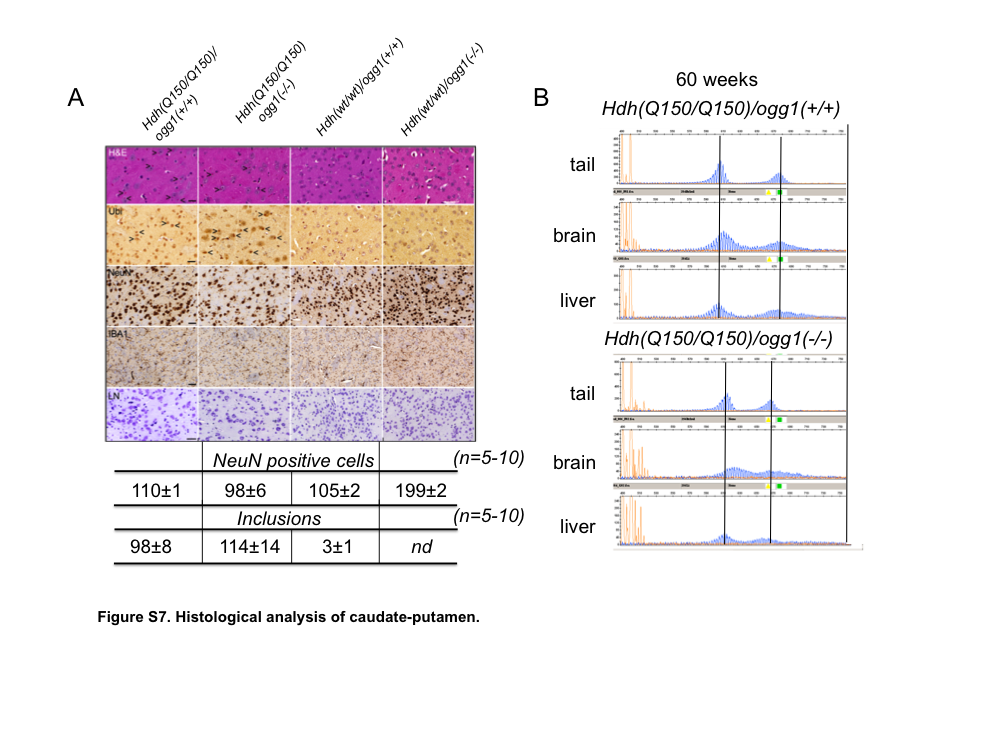

Supplement: S7 Fig — (A) Histology of the caudate/putamen of Hdh(Q150/Q150)/ogg1(+/+), Hdh(Q150/Q150)/ogg1(-/-) and controls, Hdh(wt/wt)/ogg1(+/+) and Hdh(wt/wt)/ogg1(-/-) animals, around 50 weeks of age. H&E (Hematoxylin & Eosin stain), Luxol-Nissl (LN). The small black arrows indicate protein-rich inclusions. IBA1 (microgliosis marker), NeuN (neurons), and ubiquitin (Ubi), Black arrows indicate inclusions, as stated in text. Scale bar is 50μm except for Ubi staining which is 100μm. Genotypes are indicated. Quantification of neurons by NeuN staining comprised 3 animals, 5–10 tissues slices and 10 random fields on each slice. (B) One example showing scans in which expansion was larger in some tissues in whole brain in Hdh(Q150/Q150)/ogg1(-/-) relative to Hdh(Q150/Q150)/ogg1(+/+) and animals at 60 weeks. Expansion in both lines is similar. Examples of expansion distribution in individual mice in tail, brain, and liver, as indicated. Size markers are indicated in orange. (TIF) [file pgen.1005267.s007.tif]

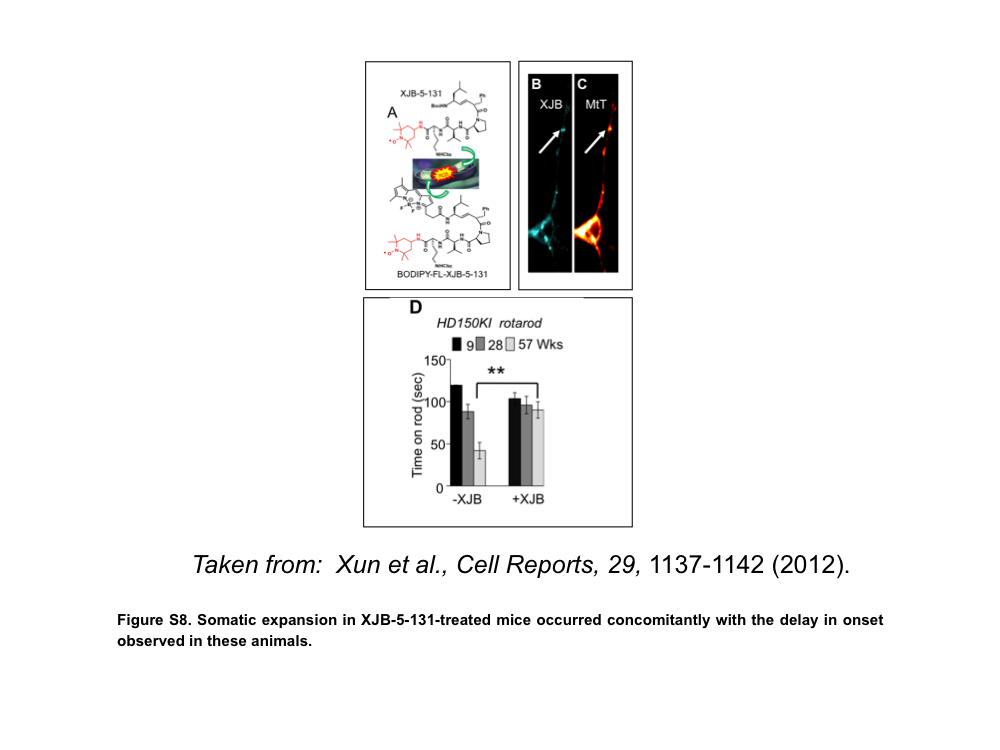

Supplement: S8 Fig — Tissue was taken from Hdh(Q150/Q150) animals reported by Xun et al., 2012, and tested for expansion here (Fig 5). (A) Structure of XJB-5-131 and its fluorescent derivative BODIPY-FL-XJB-5-131. The tempol radical scavenger portion (red), and the MT targeting moiety gramicidin S (black) are indicated. (B and C) MT staining with BODIPY-FL-XJB-5-131 co-localizes in primary neurons with Mitotracker, a mitochondrial dye (MtT). (D) Suppression of motor decline by treatment. Time on the rod decreases in Hdh(Q150/Q150) (here labelled as HD150KI) animals and is restored in animals after 9, 28, and 52-weeks of treatment with XJB-5-131. (TIF) [file pgen.1005267.s008.tif]
